# Development of cognitive, motor, metabolic, and mutant huntingtin aggregation in the zQ175 mouse model of Huntington’s disease

**DOI:** 10.1038/s41598-025-17956-5

**Published:** 2025-10-03

**Authors:** Fiona H. McLean, Olivia Monteiro, Mariah J. Lelos, Thanapon Ekkunagul, Rachel M. Spicer, Jonas Rybnicek, Jeremy J. Lambert, Rosamund F. Langston

**Affiliations:** 1 Diabetes, Endocrinology and Reproductive Biology, University of Dundee, Ninewells Hospital & Medical School, Dundee, DD1 9SY UK; 2 Neuroscience, University of Dundee, Ninewells Hospital & Medical School, Dundee, DD1 9SY UK; 3 Faculty of Medicine, Macau University of Science and Technology, Macau, China; 4 Brain Repair Group, School of Biosciences, Cardiff University, Cardiff, CF10 3AT UK; 5 Division of Diabetes, Endocrinology and Metabolism, Vanderbilt University Medical Center, 2213 Garland Avenue, Nashville, TN 37232 USA; 6 Diabetes Endocrinology and Reproductive Biology, University of Dundee Ninewells Hospital and Medical School, Dundee, DD1 9SY UK

**Keywords:** Neuroscience, Huntington's disease, Neurodegeneration

## Abstract

Huntington’s disease (HD) is an inherited neurodegenerative disease. In humans, the clinical diagnosis is often dependent on the emergence of motor symptoms. However, cognitive impairments and metabolic changes can be early indicators. HD mouse models are a useful tool to understand disease progression, however, relatively few studies have monitored the timeline for the emergence of cognitive indices with motor and metabolic phenotypes in parallel. In this study, cognitive, motor, and metabolic phenotypes were investigated at different ages in the zQ175 knock-in mouse alongside immunohistochemical and long-term potentiation (LTP) studies. We demonstrated that zQ175 mice developed impaired hippocampal LTP at 3-months and cognitive deficits in visuospatial attention were evident by 4-months. Long-term and spatial memory impairments emerged by 12-months, alongside motor impairments. Additionally, an anxiolytic-like phenotype emerged at 6-months. Differences in body weight were also detected from 6-months onwards, primarily driven by a reduction in fat mass. Additionally, reduced brain weight and the presence of huntingtin aggregates in the hippocampus, striatum and hypothalamus were observed at 12-months. These data support the zQ175 mouse as a model of HD, which recapitulates many aspects of the disease progression in humans and can be used to understand mechanisms underlying the disease.

**Supplementary Information:**

The online version contains supplementary material available at 10.1038/s41598-025-17956-5.

## Introduction

Huntington’s disease (HD) is an inherited autosomally dominant, neurodegenerative disease, caused by a mutation in exon 1 of the *huntingtin* (*HTT*) gene^1^. This mutation results in an expansion of a CAG repeat, which encodes a polyglutamine tract in the amino-terminus of the huntingtin protein. The severity of the disease progression and life expectancy have been shown to be inversely correlated with the number of CAG repeats, meaning the higher the number of CAG repeats, the earlier the onset of symptoms^2^. Individuals with 35 or less CAG repeats do not develop HD. An intermediate range of approximately 36–39 CAG repeats may result in the individual producing HD offspring or developing HD in later life^3^. The presence of 40 or more CAG repeats will result in the manifestation of the disease, with individuals with 55 or more CAG repeats sometimes showing symptoms in childhood and teenage years^4^.

Symptoms of HD encompass a triad of motor, psychiatric and cognitive symptoms, which become more debilitating as the disease progresses. The most well characterised symptom is the development of involuntary movements and incoordination known as chorea. As the disease progresses, chorea declines and immobility motor symptoms, including dystonia, rigidity and bradykinesia, become more prominent^5^. HD patients also display metabolic symptoms including unintended, catabolic weight loss and altered endocrine system regulation^6^. As our understanding of HD has improved, it has been established that cognitive and psychiatric symptoms occur in the prodromal phase of the disease, sometimes more than 10 years prior to the onset of motor symptoms^7^. HD patients can suffer from a myriad of cognitive symptoms including learning and memory impairments, as well as difficultly in planning, initiating, adapting, concentrating and multitasking, whilst psychiatric symptoms include anxiety, depression, obsessive-compulsiveness and psychosis^5,7,8^. There is a high prevalence of these debilitating symptoms early in the disease progression, with mild-cognitive impairment (MCI) present in the prodromal phase of 40% of patients^7^. At the onset of motor symptoms, MCI is present in 84% and dementia in 5% of patients^8^. In a 5 year follow up, the number of patients suffering from dementia had increased to 69%. Patients and carers report cognitive and psychiatric symptoms as some of the most life-limiting, as they lead to behavioural dysfunction, which can prevent patients from utilising motor abilities still available to them, even in the prodromal stage of the disease^9^. Historically, there has been a focus on the motor symptoms, which has led to gaps in knowledge surrounding the cognitive manifestation of the disease, cognitive aspects of HD mouse models and the development of therapeutics to treat cognitive symptoms.

Several mouse models with CAG expansions have been developed to emulate the human disease, and these models fall into the category of either transgenic or knock-in models. Transgenic models contain either a fragment or a full-length copy of the human *Htt* gene with expanded CAG repeats of varying lengths, which is inserted randomly into the mouse genome, and therefore expressed alongside the endogenous huntingtin protein (e.g., ‘R6’, ‘HD’, ‘YAC’ and ‘BAC’ mouse lines)^10,11^. These models can have several caveats including absence of HD symptoms, accelerated development of symptoms shortening the experimental study window and low huntingtin levels. Whilst transgenic mouse models are useful, in many respects, they can have limitations which reduce their translational value.

Knock-in (KI) models contain expanded CAG repeats of varying length, which have been inserted into the endogenous mouse *Htt* gene. KI models permit the generation of heterozygous models, with one wild-type allele and one allele containing the CAG expansion, which can allow for expression patterns to be closer to that in humans as homozygosity is very rare in humans^12^. Such mouse lines exist with a range of CAG repeat lengths available, from 20 to 365, although it should be noted CAG repeats of more than 80 are uncommonly seen in adult-onset HD^4^.

The zQ175 KI is an HD model that was developed from an unstable expansion in the CAG region of the CAG 140 KI model^13,14^. This has resulted in the zQ175 mouse containing a knock-in allele which has the mouse *Htt* exon 1 replaced by the human HTT exon 1 sequence with a 190 CAG repeat tract. This model is of particular interest, as it is similar to the CAG 140 KI model, which has been more widely studied, except it has more CAG repeats, therefore studying the zQ175 mouse can provide information on CAG repeat length and the impact on disease progression. Variants of this model have also been established, in which the cassette used to generate the model has been excised^15,16^.

**Fig. 1:**
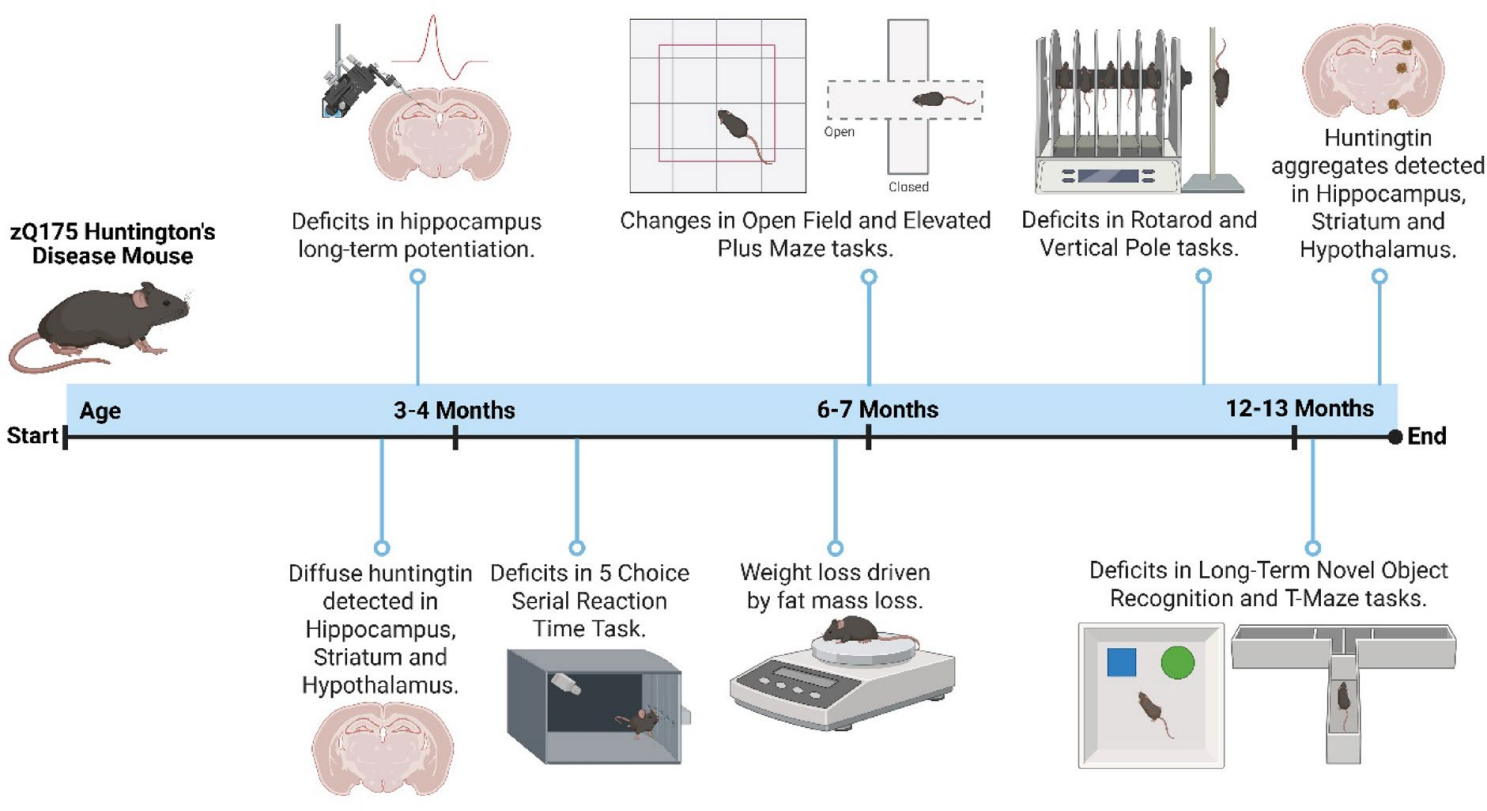
Schematic timeline of zQ175 mouse phenotype. Created in BioRender. McLean, F. (2025) (https://BioRender.com/d9uxcba).

In this study we have characterised the phenotypic progression of the zQ175 mouse from 3 to 13 months old (Fig. 1). We used a bank of cognitive and psychiatric behavioural assays to assess the severity of symptoms seen early in human HD, which are often understudied in HD mouse models. Additionally, we measured metabolic and motor symptom manifestation to explore associations of psychiatric and cognitive outcomes against metabolic and motor data. Immunohistochemical staining was performed to analyse mutant huntingtin protein aggregation in brain regions associated with relevant behavioural, metabolic, and motor observations. Furthermore, electrophysiology was carried out to investigate hippocampal long-term potentiation (LTP), which is a form of synaptic plasticity often associated with learning and memory^17^. The overall aim of this study was to provide a phenotype timeline to help inform when the zQ175 mouse displays particular HD traits. Our study provides new information about the heterozygous zQ175 mouse line, particularly cognitive characteristics, and aligns these to changes in motor abilities and metabolic aberrations.

## Materials and methods

### Experimental design

All studies adhered to UK Home Office regulations according to the Animals (Scientific Procedures) Act, 1986, were in accordance with the European Directive on the Protection of Animals used for Scientific Purposes 2010/63/E and followed ARRIVE guidelines. Experimental protocols were approved by the University of Dundee Ethical Review Committee and the Cardiff University Ethics Committee. Male B6J.129S1-Htttm1Mfc/190ChdiJ heterozygous mice (Stock number: 027410, Jax) were bought in and bred in house with female C57Bl6/J mice to produce mixed litters of wild-type and heterozygous offspring. Following this, the colony was maintained through the breeding of heterozygous females and wild-type males to minimise genetic anticipation^18^. Experimental mice were group housed with different genotypes housed together. All mice were maintained on 12:12 h light: dark cycles with access to food and water *ad libitum*. All behavioural tasks were conducted during the light phase. Experimenters were blind to the genotype during testing. Environmental enrichment was provided in the form of red tubes, cardboard houses, and wooden chew sticks. No mice died prematurely, and no seizures observed. Information on mouse cohort numbers, genotypes in each group, sex, age, and what cohorts were used for each behavioural test or technique can be found in Supplementary Information (Supplementary Table S1).

## Genotyping

Ear clips were analysed by Transnetyx, USA. A random selection of samples was verified in house. Ear biopsies were collected from mixed litters and DNA was extracted using a NucleoSpin Tissue Mini kit for DNA from cells and tissue (740952.50, Machery and Nagel GmbH & Co.) following the manufacturer’s guidelines. Purified DNA was used as templates in a standard PCR using the GoTaq G2 Hot Start Master Mix (Promega) to detect a mutant DNA band of 280 bp and a control wild-type band of 206 bp when separated on a 1% agarose gel. Primer details are as follows: mutant forward CTTGGGTGGAGAGGCTATTC; mutant reverse AGGTGAGATGACAGGAGATC; control forward CAAATGTTGCTTGTCTGGTG; control reverse GTCAGTCGAGTGCACAGTTT.

## Body weight, brain weight and body composition measurements

Body weight was measured monthly. Body composition was measured using an Echo MRI 2011 Body Composition Analyser, version 11.0413.0927 (Echo Medical Systems LLC, US). For brain weight measurements, whole brains were dissected including olfactory bulbs and cerebellum, and weighed.

### 5 choice serial reaction time task (5CSRTT)

*Training for the 5CSRTT consisted of an initial training phase to teach the mice to retrieve rewards from the magazine (magazine training)*,* followed by training to learn to nosepoke into the operandum embedded within the 5 choice array (fixed response training). Thereafter*,* training on the 5CSRTT itself commenced.*

#### Magazine training

**Fig. 2:**
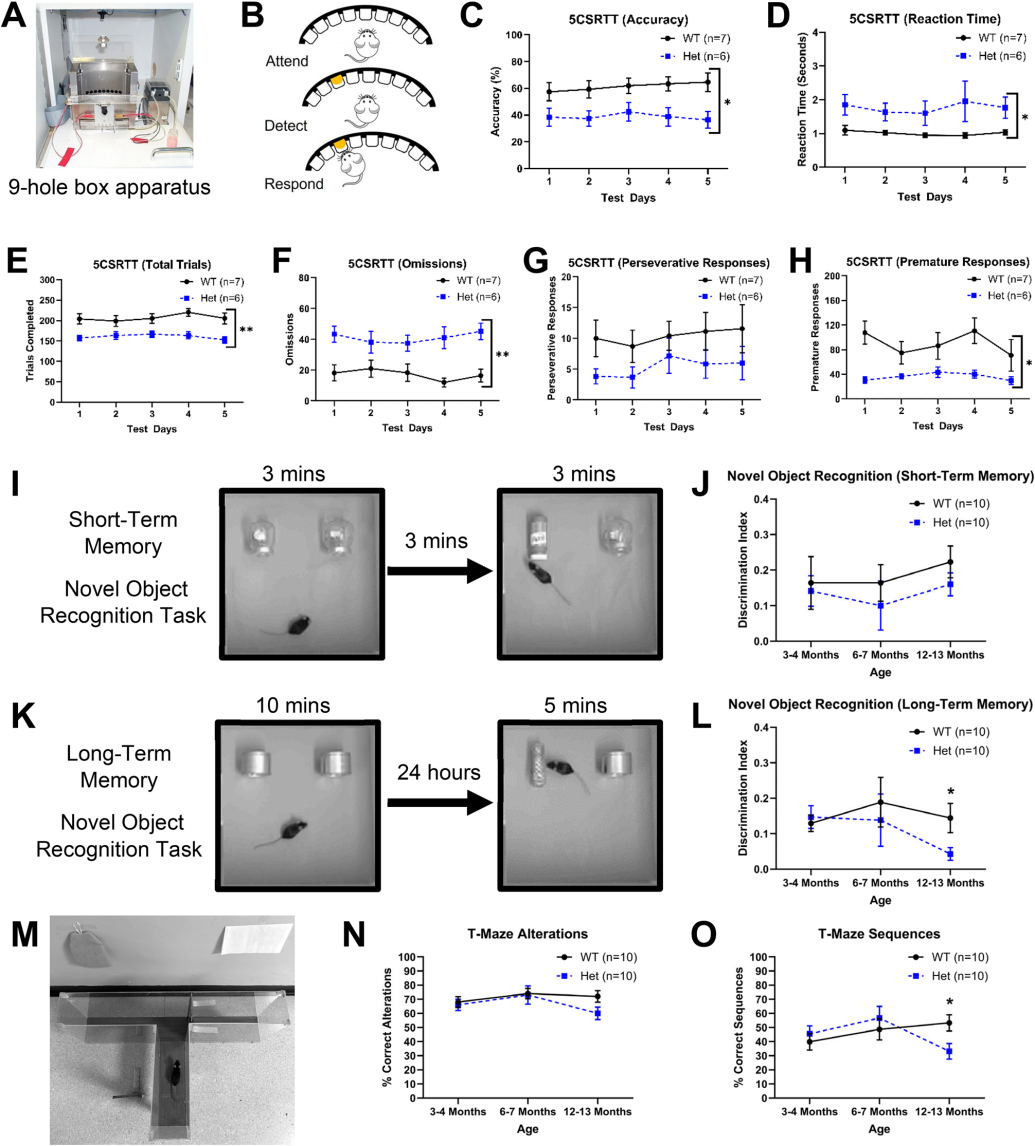
Cognitive tasks in wild-type and heterozygous zQ175 mice. (**A**) Representative image of 9-hole box apparatus for operant testing. (**B**) Schematic image depicting the basic requirements for response accuracy in the 5CSRTT. Mice attended the 9-hole array then detected a stimulus light that flashed for 1 second. Following this, mice were required to nose poke in the hole to obtain a reward. (**C**) Response accuracy was impaired in Het mice relative to WT. (**D**) Reaction time was impaired in Het mice relative to WT. (**E**) Het mice completed fewer trials than WT mice. (**F**) Het mice omitted responses more often than WT mice. (**G**) There were no differences in the rate of perseverative responding between WT and Het mice. (**H**) Het mice responded prematurely significantly less often than WT mice. (**I**) Short-term memory novel object recognition task graphic. (**J**) Short-term memory novel object recognition task graph. There were no differences in performance between WT and Het mice at 3-4, 6-7 or 12-13 months. (**K**) Long-term memory novel object recognition task graphic. (**L**) Long-term memory novel object recognition task graph. There were no differences in performance between WT and Het mice at 3-4 or 6-7 months. At 12-13 months, Het mice showed a deficit in task performance. (**M**) T-Maze graphic. (**N**) T-Maze alternations graph. There were no differences in performance between WT and Het mice at 3-4 or 6-7 months. At 12-13 months, Het mice showed a slight deficit, although this was not significant. (**O**) T-Maze sequences graph. There were no differences in performance between WT and Het mice at 3-4 or 6-7 months. At 12-13 months, Het mice showed a significant impairment. Two-way repeated measures ANOVA and pairwise comparisons with Greenhouse–Geisser and Bonferroni corrections where appropriate. Data are mean ± SEM.  WT vs Het *p <0.05, **p <0.01, ***p <0.001. (C-H) WT *n*=7, Het *n*=6; (J, L, N, O) WT* n*=10, Het *n*=10. Wild-type (WT) and heterozygous (Het).

At 3.5 months of age (14 weeks), mice were introduced to the 9-hole operant chambers (Fig. 2A + B) and magazine training commenced. On day 1, the peristaltic pump connected to the magazine delivered a 10 s administration of reward (strawberry Yazoo milkshake) in the food hopper. Over this 20 min session, the house light remained off and the magazine light illuminated, to enable the mice to associate the light with the reward. On days 2 and 3, the magazine light was illuminated and a 30 µl reward was delivered simultaneously over a 20 min session.

#### Fixed response training

The central hole of the 9-hole array was illuminated and mice were taught to nose poke in return for a 30 µl reward. Once > 50 correct responses were obtained, the mice were moved to the next phase. Our standard criteria dictate that if mice did not reach these criteria, they were considered trained once their performance reached asymptote. However, in this study, all mice executed > 50 responses during this training phase.

#### 5CSRTT

For the initial training, the light stimulus was presented in one of the 5 holes of the array for an unlimited duration. The stimulus was terminated and 30 µl reward was presented upon nose poke in the illuminated hole. Incorrect nose pokes were not punished. Once responding in all holes was achieved, mice were presented with a 10 s light stimulus which was rewarded upon correct nose poke. Incorrect nose pokes or no response within 10 s resulted in a ‘time out’ phase in which the house light was illuminated for 5 s. The stimulus duration was subsequently reduced to 5 s for 3 days followed by a 2 s stimulus for 5 days. Mice were trained on the final 5-CSRTT with a 1 s stimulus duration for 5 days. They were subsequently tested for a further 5 days on the 5CSRTT with a 1 s stimulus and these data were analysed for genotype effects.

## Short-term and long-term novel object recognition tasks

The testing arena was 40 × 40 × 40 cm. Dual Lock (3 M, Bracknell, UK) adhesive strips were secured at two positions on the floor 10 cm from the wall furthest from the experimenter, 10 cm from the edge of the arena and 20 cm apart. Objects, composed of non-porous substances (e.g. glass, plastic or metal) and a maximum of 12 cm in any dimension, were attached to the Dual-Lock strips. The short-term novel object recognition task consisted of a 3-minute sample phase, 3-minute interval and 3-minute test phase (Fig. 2I). The long-term novel object recognition task consisted of a 10-minute sample phase, 24-hour interval and 5-minute test phase (Fig. 2K). In the sample phase, two identical objects were presented to the mouse. In the test phase, these objects were replaced with two new objects, one which was identical to the objects in the sample phase and one novel object. Between phases and different mice, the arena was wiped down with lemon-scented anti-bacterial wipes. Object exploration was defined as actively engaging with an object, including sniffing, whisking and close inspection within ~ 2 cm. Sitting on/near the object without engaging or compulsive chewing of an object was not classed as exploration. Four mice were tested simultaneously in identical set-ups to reduce variation resulting from time of day. Data were excluded if the mouse explored each object in the sample phase for less than 5 s or both objects in the test phase for a total time of less than 10 s. The time spent exploring was converted into a discrimination index (DI) calculated using the formula: (time spent exploring novel object – time spent exploring familiar object)/(time spent exploring novel object + time spent exploring familiar object). Use of this formula normalises data between animals. A DI of greater than 0 indicates a memory of the familiar object. Data were blind scored manually. Each mouse carried out 2 trials of both the short-term and long-term tasks, which were balanced for side and novel object bias. An average of the 2 trials was calculated for each mouse.

## T-Maze

The maze consisted of 30 cm high transparent walls, a 40 cm long start arm, and 30 cm long left and right choice arms (Fig. 2M). All arms were 10 cm wide. External visual cues were fixed to the wall beside the left and right choice arms. For the first trial, one of the arms was blocked off using a panel, only allowing the mouse to be able to explore either the left or right arm. This was counterbalanced across the groups of mice to account for side bias. Mice were placed into a 10 × 10 cm start area in the start arm, which was blocked off using a panel. After 10 s, the panel was removed, and the mouse was allowed to explore the accessible arm for 3 min. The mouse was then encouraged back to the start area with a plastic spatula to avoid the experimenter picking up the mouse. The panel was then replaced, and the mouse was left in the start area for 10 s. The panel was then removed, and the mouse was allowed to choose either the left or right arms. A choice was only made when the body of the mouse, including the tail, had entered an arm. The other arm was then blocked off using a panel and the mouse was allowed to explore the chosen arm for 1 min. After the minute, the mouse was returned to the start area, as described previously, and the trial was repeated. A total of 10 choice trials, not including the first trial, were carried out. The arm choice for each trial was recorded, as well as the latency to make that choice. A ‘correct alternation’ was recorded when a mouse selected the alternate arm to the one in the previous trial (e.g. left then right arm chosen). A ‘correct sequence’ was recorded when a mouse carried out 3 consecutive correct alternations (e.g. left then right then left arm chosen). The maze was wiped with lemon-scented anti-bacterial wipes between mice.

### Burrowing task

**Fig. 3:**
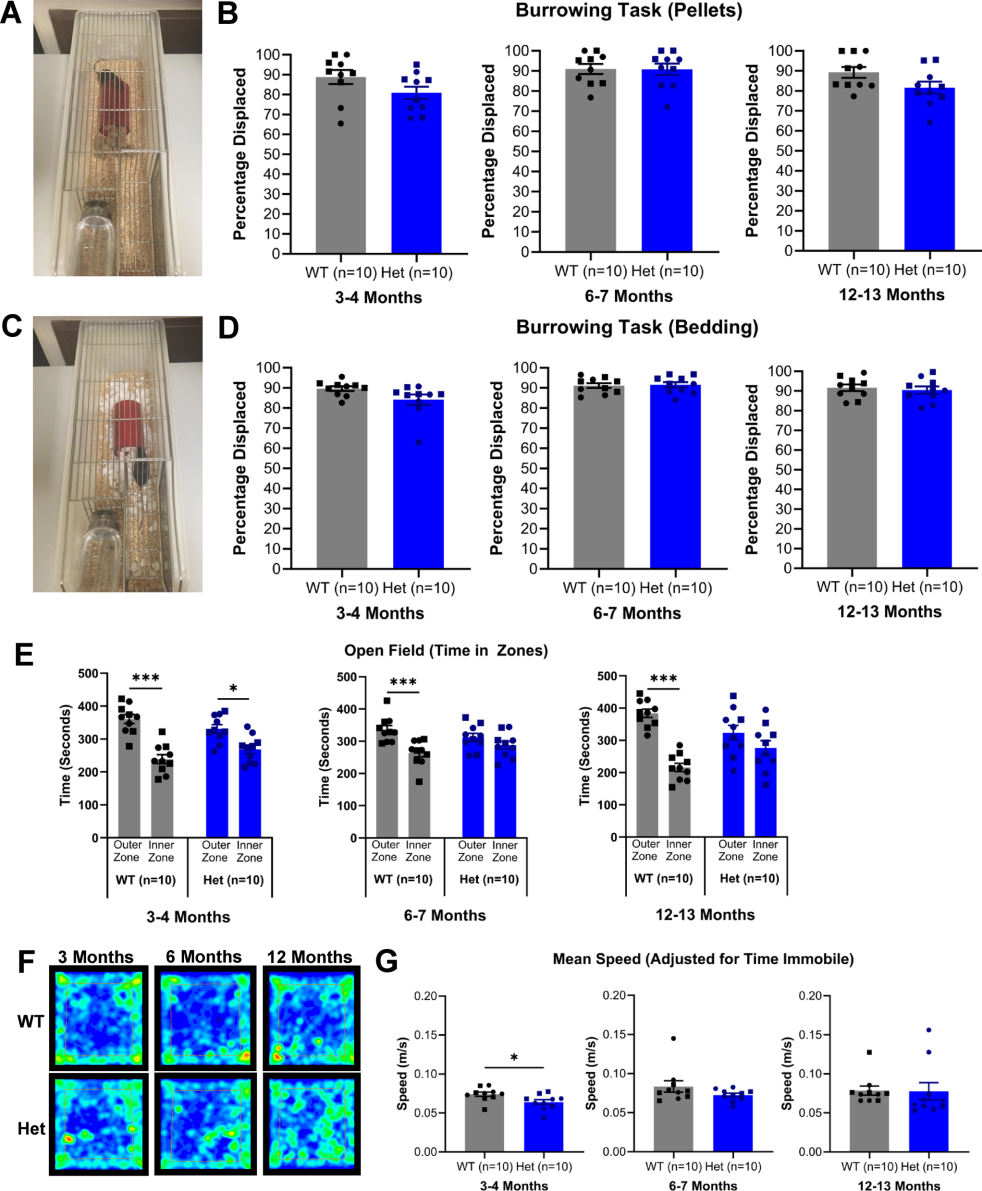
Psychiatric tasks in wild-type and heterozygous zQ175 mice. (**A**) Image of burrowing task with pellets. (**B**) Burrowing task with pellets. There were no differences detected between WT and Het mice. (**C**) Image of burrowing task with bedding. (**D**) Burrowing task with bedding. There were no differences detected between WT and Het mice. (**E**) Open field. At 3–4 months, WT mice spent more time in the outer zone than the inner zone and Het mice showed a similar phenotype. At 6–7 months, WT mice also spent more time in the outer zone than the inner zone, however Het mice displayed an anxiolytic-like phenotype and did not spend significantly more time in either zone. At 12–13 months, WT mice continued to spend more time in the outer zone than the inner zone, however, as seen at 6–7 months, Het mice displayed an anxiolytic-like phenotype and did not spend significantly more time in either zone. (**F**) Example heat maps of open field for WT and Het mice at 3–4, 6–7 and 12–13 months. WT mice at 3–4, 6–7 and 12–13 months and Het mice at 3–4 months were tracked round the outside and corners of the arena more, whereas Het mice at 6–7 and 12–13 months were tracked more in the centre of the open field. (**G**) Speed in open field. There was a difference between WT and Het mice in speed in the open field at 3–4 months, however not at 6–7 or 12–13 months. One-way or two-way ANOVA with Bonferroni correction where appropriate. (B, D, E, G). Data are mean ± SEM. Symbols are square (male) and circle (female). WT vs. Het *p < 0.05, **p < 0.01, ***p < 0.001; All groups *n* = 10. Wild-type (WT) and heterozygous (Het).

Mice were placed individually in a clean cage with sawdust, access to water, and a 10 cm x 6 cm x 6 cm red tube filled with either chow pellets (Fig. 3A) or white paper bedding (Fig. 3C). The white paper bedding was used in addition to the pellets to control for possible motor impairments in the heterozygous zQ175 mice, since it is a comparatively light material. The mice were left for 1 h. The tube was weighed at the beginning and end of the hour, allowing for displaced material to be calculated.

## Open field

Arena was 40 × 40 × 40 cm with an outer zone of 5 cm from the arena walls and an inner zone of 30 × 30 cm. Data was recorded using AnyMaze software (Stoelting Co., Illinois, USA). Mice were placed in the arena, facing the wall closest to the experimenter, and given 10 min to freely explore. AnyMaze automatically recorded the time spent in each zone, the total distance travelled and the average speed of the mouse. Four mice were tested simultaneously in identical set-ups to reduce variation from time of day.

## Elevated plus maze

Maze was 95 cm off the ground and consisted of 4 arms 5 cm x 30 cm. The neutral zone where the arms meet was 5 × 5 cm. Open arms were opposite each other and had no walls whilst the closed arms had walls at a height of 20 cm. Data was recorded using AnyMaze software (Stoelting Co., Illinois, USA). Mice were placed in neutral zone and given 10 min to freely explore. AnyMaze automatically recorded the time spent in each arm as well as the total number of entries into each arm.

### Vertical pole

**Fig. 4:**
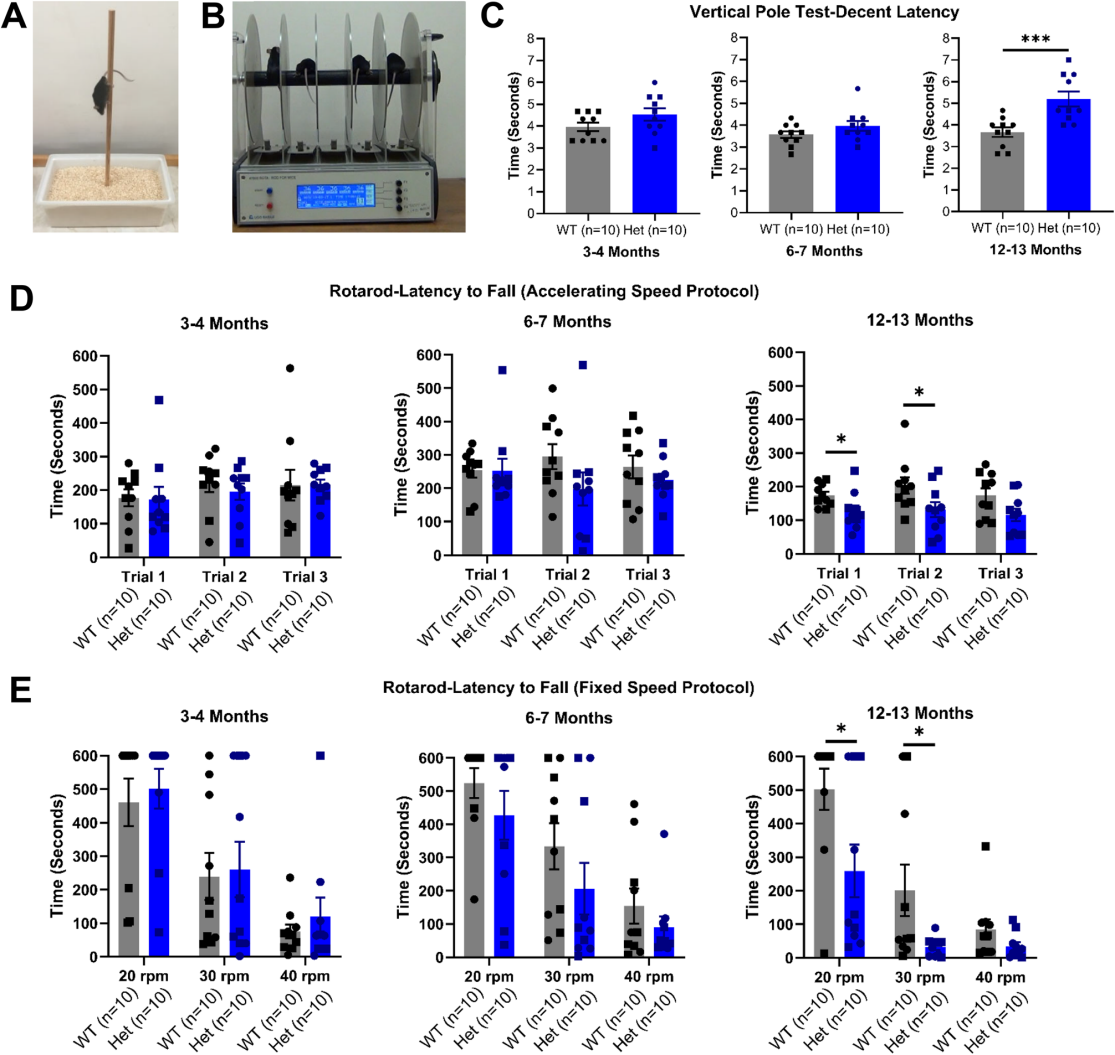
Motor tasks in wild-type and heterozygous zQ175 mice. (**A**) Vertical pole test graphic. (**B**) Rotarod graphic. (**C**) Descent latency in vertical pole test. There were no differences between WT vs. Het at 3–4 or 6–7 months. However, Het mice took longer to descend the pole at 12–13 months. (**D**) Latency to fall from rotarod with accelerating protocol. There were no differences at 3–4 or 6–7 months between WT and Het mice. At 12–13 months there were significant difference between WT and Het mice. (**E**) Latency to fall from rotarod with fixed speed protocol. At 3–4 or 6–7 months there were no differences between WT and Het mice at 20 rpm, 30 rpm or 40 rpm. At 12–13 months there were differences between WT and Het mice at 20 rpm and 30 rpm but not 40 rpm. One-way ANOVA or two-way repeated measures ANOVA with pairwise comparisons and Greenhouse–Geisser and Bonferroni corrections where appropriate. (C-E) Data are mean ± SEM. Symbols are square (male) and circle (female). WT vs. Het *p < 0.05, **p < 0.01, ***p < 0.001; All groups *n* = 10. Wild-type (WT), heterozygous (Het) and rotations per minute (rpm).

The wooden vertical pole was 40 cm tall and was fixed in a plastic box 35 × 16 cm filled with sawdust (Fig. 4A). Mice underwent 2 training sessions with 2 trials in each session. In each trial, mice were placed at the top of the pole and the length of time the mouse took to climb down was recorded. The test session consisted of 3 trials which were averaged. There was approximately an hour between sessions and training and test sessions were carried out on the same day.

### Rotarod

Mice were tested on an accelerating protocol and a fixed speed protocol on an automated rotarod (Fig. 4B). For the accelerating protocol, mice were tested in three 600-second trials with an accelerating speed from 6 to 40 rpm. For the fixed speed protocol, mice were tested in three 600-second trials with fixed speeds of 20, 30 and 40 rpm. The latency to fall from the rod was recorded. Mice were not disqualified for holding on to the rotarod and rotating. If mice remained on the rod for more than 600 s, they were removed, and a score of 600 s was recorded. Results were also analysed with an adjustment made for the weight differences between wild-type and heterozygous mice by multiplying time on the rotarod by a factor (individual animal weight/average zQ175 weight) (Figure S1)^15^.

### Determination of huntingtin aggregation in the brain

#### Perfusions

Mice were humanely euthanised at 3–4, 6–7 and 12–13 months old with 0.2 ml per 30 g body weight of 200 mg pentobarbital sodium (Euthatal, Merial, UK) via intraperitoneal injection. Depth of anaesthesia was assessed using loss of toe pinch and corneal reflex. Once the mice were unresponsive, they were transcardially perfused with 30 ml of cold 0.9% phosphate buffered saline followed by 50 ml of 4% paraformaldehyde (PFA) in 0.1% phosphate buffer using a peristaltic pump. The brains were removed and post-fixed overnight in 4% PFA in 0.1% phosphate at 4 °C. Following this, brains were incubated in 2% PFA in 0.1% phosphate and 15% sucrose overnight at 4 °C. Subsequently, brains were then cryoprotected by incubating in 30% sucrose solution at 4 °C for a minimum of 24 h until saturated. Brains were snap-frozen and embedded in TissueTek. Coronal sections of 20 μm were cut using a cryostat and stored as free-floating sections in 0.01% sodium azide solution until immunohistochemical staining.

### Immunohistochemistry

All steps were carried out with tissue sections free-floating in round, glass staining containers using compartment nets with 8 segments. Each net was counterbalanced across ages and genotype. Incubations used an orbital rotator wherever possible. Sections were removed from 0.01% sodium azide solution and washed 3 × 5 min in 1 x PBS. Antigen retrieval was carried out by incubating sections for 10 min at 95 °C in citric acid buffer (0.01 M, 0.05% Tween 20, pH 6.0). Citric acid buffer was pre-heated using a microwave and temperature was maintained using a water bath. Subsequently, sections were left to cool gradually for 15 min before being washed 3 × 5 min in 1 x PBS. Endogenous peroxidase activity was then blocked by incubating sections in 3% hydrogen peroxide in methanol for 5 min. Sections were washed 3 × 5 min in 1 x PBS-Triton (0.3%) and then blocked for non-specific protein binding with 3% normal goat serum in 1 x PBS-Triton (0.3%) for 1 h at room temperature. Sections were then incubated overnight at room temperature with EM48 primary antibody (MAB5374, Millipore; RRID: AB_177645) at 1:100 dilution, or S830 (kind gift from Professor Gill Bates) at 1:2000, in 1% normal goat serum in 1 x PBS-Triton (0.3%). Sections were washed 3 × 5 min in 1 x PBS-Triton (0.3%) followed by incubation in biotinylated goat-anti-mouse IgG (Vectastain Elite ABC kit, VECTOR, PK4002) at 1:200 dilution in 1% normal goat serum in 1 x PBS-Triton (0.3%) for 2 h at room temperature. Sections were washed 3 × 5 min in 1 x PBS-Triton (0.3%), followed by 5 × 3 min in 1 x PBS. ABC solution was prepared according to kit instructions and sections were incubated for 1 h at room temperature. Sections were washed 5 × 3 min in 1 x PBS. DAB Peroxidase (HRP) Substrate Kit (VECTOR, SK-4100) was used to develop reaction (72 drops of buffer stock:144 drops DAB stock:72 drops of hydrogen peroxide) for approximately 3 min at room temperature. Sections were washed 5 × 3 min times in dH2O and then mounted onto glass slides. Once slides were dry, coverslips were mounted using DPX Mountant (SIGMA, 06522-100 ml).

### Image acquisition and analysis

Manual, brightfield microscopy image capture was conducted using the ZEISS Axioskop 2 MOT(Motorized) fluorescence microscope with Axiocam Color 431 − 312 camera (Carl Zeiss Microscopy GmbH) and Axiovision rel. 4.8.2 SP2 Software (Carl Zeiss Microscopy GmbH). 5x and 40x Plan Neofluar objectives were used, with pixel scaling at 2.127 and 0.2662 μm per pixel respectively. Three regions of interest were analysed; Hippocampus (Dorsal): −1.855 mm to −2.055 mm; Striatum (Caudoputamen): −0.245 mm to −0.02 mm; Hypothalamus (Lateral Hypothalamic Area) −1.255 mm to −1.455 mm. Aggregations were counted manually using ImageJ software version 1.52 (National Institutes of Health, USA). A sampling area overlay measuring 65 × 155 μm (a surface area of ~ 0.1mm^2^) was applied to regions of interest across 40x objective images for consistent aggregate quantification. Each visible aggregate was individually marked and automatically counted by the multi-point tool marker. Data was analysed across regions from 1 slice from 3 individual mice per group. For the hippocampal proper, an average of the subregions was taken before averaging across the group.

### In vitro recording of field excitatory post-synaptic potential

Preparation of mouse hippocampal brain slices for extracellular recordings were as described previously^19^. With the experimenter blinded to the genotype of the mice, heterozygous zQ175 mice and wild-type litter mates aged 1 month or 3 months old were decapitated following cervical dislocation, and their brains quickly removed and placed in “ice-cold”, oxygenated (95% O_2_, 5% CO_2_) artificial cerebrospinal fluid (aCSF) (composition in mM: 124 NaCl, 3 KCl, 1.75 MgCl_2_, 1 CaCl_2_, 1.25 NaH_2_PO_4_, 26 NaHCO_3_, and 10 D-glucose) with the osmolarity of the aCSF adjusted to 300–310 mOsm. Brain slices (400 μm) containing sagittal sections of the hippocampus were prepared with a VT1000E tissue slicer (Leica). Following a recovery period of 1 h in the oxygenated aCSF, a single slice was transferred to a recording chamber (Scientific Systems Design, Mississauga, Ontario, Canada), where it was submerged and continually perfused (flow rate ~ 2 ml/min) with an oxygenated modified aCSF that now contained 1 mM MgCl_2_ and 2.5 mM CaCl_2_. The gravity feed was manipulated to maintain a constant flow rate and a Gilson pump (AD Instruments, model Minipuls evolution, Calgrove, Oxfordshire, UK) was used for suction to remove aCSF from the recording chamber. To monitor basal synaptic transmission a bipolar stimulating electrode, made from twisted Teflon-coated tungsten wire (Advent research materials, Ltd, Eynsham, Oxfordshire, UK), was used to stimulate the Schaffer collateral-commissural pathway from area CA3 to the CA1 region of the hippocampus. The stimulus was delivered to the slice once every 30 s to record dynamic changes in the neurally-evoked field excitatory postsynaptic potential (fEPSP). The stimulus was delivered by a constant current isolated electronic stimulator (Digitimer Ltd, Hertfordshire, UK), which was electrically isolated to prevent interference from the mains electrical noise. Additionally, the signal was fed through a “Hum Bug” (Digitimer Ltd, Hertfordshire, UK) to further reduce electrical interference. The stimulatory current was adjusted to produce a response with a fEPSP slope that was 40% of the maximum population spike-free response. The fEPSPs were recorded using an aCSF-filled glass borosilicate microelectrode, (Kind precision glass, Inc., Claremont, USA), which had a resistance of less than 5 MΩ (inner diameter of 0.69 mm) and was placed in the apical dendritic layer of the CA1 pyramidal cells. Within the recording electrode was a silver chloride wire (Advent research materials, Ltd, Eynsham, Oxfordshire, UK) and this, together with a ground electrode of silver chloride wire, was attached to an isolated differential amplifier, where the signal was amplified and filtered (10 kHz) (Warner Instrument Corporation, Connecticut, USA). The output from the amplifier was fed through an oscilloscope (Tektronix, Oregon, USA) for online monitoring of the fEPSPs and then digitized through an acquisition board (BNC-2090, National Instruments, Berkshire, UK). Both the stimulating and recording electrodes were positioned using micromanipulators under visual guidance through a microscope (Olympus, SZ30, Essex, UK). For control long-term potentiation (LTP) experiments the fEPSP resulting from a stimulus delivered 1/30 sec was monitored for 10 min to ensure stability of the recording (fEPSP slope and amplitude) prior to inducing LTP. To subsequently induce control maximal LTP, a theta-burst stimulation (TBS) protocol was delivered (4 pulses at 100 Hz, repeated 10 times, at an interval of 200 milliseconds between the groups of 4 pulses; 4-TBS). We have previously established the 4-TBS paradigm to induce maximal LTP. The fEPSP measurements were monitored for an additional 60 min after delivery of the 4-TBS. Analysis of fEPSPs in each slice was performed using WinLTP software (Anderson, https://www.winltp.com/). The number of individual slices analysed per mouse for each group can be found in Supplementary Information (Supplementary Table S1).

### Data analysis and statistics

Statistical tests were run in IBM SPSS Statistics 28. Graphs were created in GraphPad Prism (Version 10.4.2). Data are presented as mean ± SEM. One-way, two-way, three-way and repeated measures ANOVA were carried out as appropriate for each data set and alpha level adjusted with Bonferroni correction to avoid an inflated Type I error. When using repeated measures ANOVA, Greenhouse–Geisser correction was applied when there was a lack of sphericity. Sphericity was assessed by Mauchly’s sphericity test. Full statistics are reported in Supplementary Information (Supplementary Table S2). Statistics with ‘Sex’ included as a fixed factor are reported in Supplementary Information (Supplementary Table S3). No interaction was found between Genotype and Sex in any tests, with two exceptions (5CSRTT (reaction time) and open field (maximum speed)), therefore data from male and female mice of the same genotype are reported together in the main manuscript. Male and female data points are represented in scatter plots by square and circle symbols, respectively. For line graphs, male and female data is presented separately in Supplementary Figure S2.

## Results

### Cognitive deficits occur in a time relevant manner with classical symptoms of Huntington’s disease

The zQ175 mice were tested on the 5CSRTT (Fig. 2A + B). This test is sensitive to striatal dysfunction^20^ and performance on this task has been shown to be impaired in other mutant models of HD^21,22^. At 3–4 months old, heterozygous mice were less accurate on the task, which indicates impaired visuospatial attention (Fig. 2C). Their reaction time, which incorporates both an attentional and motor component, was slower in heterozygous mice (Fig. 2D). zQ175 mice also completed fewer trials (Fig. 2E) and failed to respond to the stimulus more often than wild-type mice (Fig. 2F). There was no difference in perseverative responses between the genotypes, but heterozygous mice were less impulsive, as indicated by the reduced number of premature responses (Fig. 2G + H). These data map broadly onto the human condition insofar as visuospatial dysfunction has been reported in early disease (e.g. pre-manifest stage)^23,24^. To investigate cognitive ability during disease development, different aspects of memory were tested in short-term, long-term and spatial memory tasks in mice at 3–4, 6–7 and 12–13 months old (Fig. 2I + K). Heterozygous mice performed comparably with wild-type mice in the short-term memory task at all ages (Fig. 2J). Similarly, heterozygous mice retained the ability to perform a long-term memory task at 3–4 and 6–7 months old but displayed significant deficits in this task by 12–13 months (Fig. 2L). This profile was mirrored by deficits in the spatial memory task, where heterozygous mice performed comparatively with wild-type mice at 3–4 and 6–7 months old, however, by 12–13 months old were impaired when compared with their wild-type counterparts in performing consecutive alternations to complete correct sequences (Fig. 2N + O).

HD patients frequently present with behavioural symptoms that affect everyday activities of daily living^25^. To explore if the zQ175 mouse presented an equivalent phenotype, we employed three behavioural tasks, an open field, an elevated plus maze, and a burrowing task. The burrowing task, which is designed to emulate ‘activities of daily living’ in humans, utilises the innate behaviour of a mouse to improve quality of life and provides insight into the ‘well-being’ of the mouse^26,27^. We found no significant differences between heterozygous and wild-type mice at any age (Fig. 3B). Since the removal of pellets requires a degree of motor ability, which can be impaired in HD, this task was also repeated with an alternative burrowing material in the form of light, and easy to manipulate, bedding material. When carrying out the task with the bedding material, there were also no differences between heterozygous and wild-type mice at any age (Fig. 3D). The open field paradigm provides a measurement of anxiety and revealed anxiolytic-like behaviour in the heterozygous mice at 6–7 and 12–13 months old (Fig. 3E + F)^28–30^. We observed a similar result in the elevated plus maze at 6–7 months old, with heterozygous mice spending more time in the open arm (Figure S3 A + B). However, we do not see this distinction at 12–13 months. This may be an age-related effect, as we also see a decline in time spent in the open arm in the wild-type group across all ages. In the open field, speed was measured to address any motor changes affecting performance, however, there were no differences, except at 3–4 months where heterozygous mice were slightly slower (Fig. 3G). Despite this difference, at 3–4 months heterozygous mice still presented a comparable phenotype with wild-type counterparts. The total distance travelled, maximum speed, and time immobile were also analysed, however, no differences between genotypes were found (Figure S4).

To determine if cognitive deficits coincided with classical HD symptoms, mice also underwent motor tasks at 3–4, 6–7 and 12–13 months old, designed to assess motor coordination, planning and balance. When challenged with the vertical pole task, heterozygous mice descended the pole at the same rate as wild-type mice at 3–4 and 6–7 months old, however at 12–13 months old heterozygous mice were impaired in this task as they took longer to descend the pole (Fig. 4C). Similarly, there were no differences in performance of the rotarod task in the accelerating or fixed speed protocols at 3–4 and 6–7 months (Fig. 4D + E). However, at 12–13 months old, heterozygous mice fell off quicker in the accelerating protocol, as well as at 20 rpm and 30 rpm in the fixed speed protocol (Fig. 4E). There was no difference between the groups at 40 rpm, however this is likely due to the speed being too fast for the wild-type mice to stay on long enough to show a difference between genotypes. Whilst the fixed speed protocol revealed no significant differences between genotypes at 6-months old, there is a trend showing the heterozygous mice fall off the rotarod quicker, suggesting motor impairments may start to manifest at this age. Notably, there were no differences in open-field speed at 12–13 months old in heterozygous mice, despite motor impairments seen in the rotarod at this age.

**Fig. 5:**
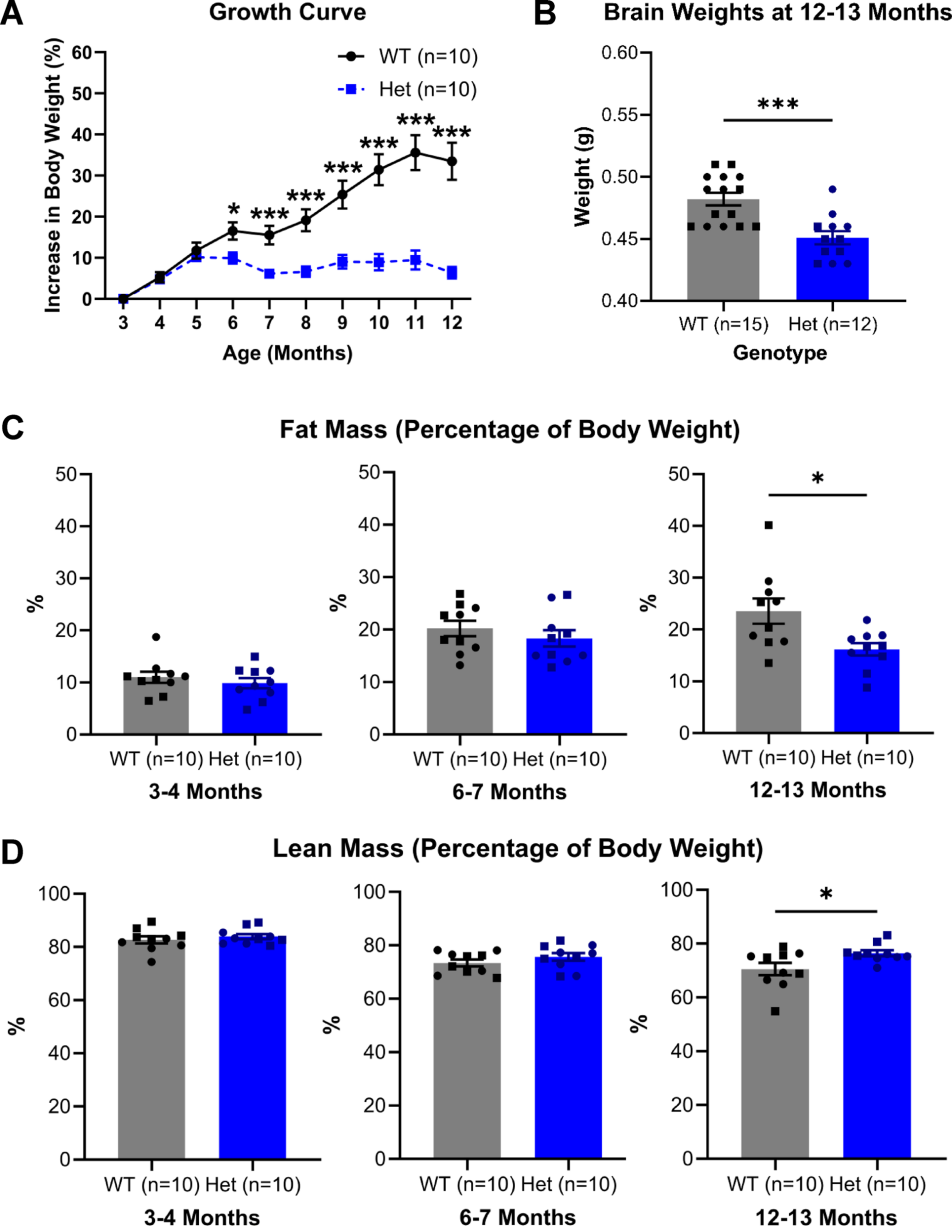
**Body weight**,** brain weight and body composition comparison of wild-type and heterozygous zQ175 mice.** (**A**) Body weight growth curve from 3 to 12 months. There were no differences between WT and Het mice at 3, 4 or 5 months. However, there were significant differences in body weight between WT and Het mice at 6, 7, 8, 9, 10, 11 and 12 months. (**B**) Brain weights at 12–13 months. Het mice had lower brain weight than WT counterparts. (**C**) Fat mass (percentage of body weight) at 3–4, 6–7 and 12–13 months. There were no differences at 3–4 or 6–7 months, however, Het mice had significantly lower fat mass than WT mice at 12–13 months. (**D**) Lean mass (percentage of body weight) at 3–4, 6–7 and 12–13 months. There were no differences at 3–4 or 6–7 months, however, Het mice had higher lean mass than WT mice at 12–13 months. One-way ANOVA or two-way repeated measures ANOVA with pairwise comparisons and Greenhouse–Geisser and Bonferroni corrections where appropriate. (A-D) Data are mean ± SEM. Symbols are square (male) and circle (female). WT vs. Het *p < 0.05, **p < 0.01, ***p < 0.001. (A, C, D) All groups *n* = 10; (B) WT *n* = 15, Het *n* = 12. Wild-type (WT), heterozygous (Het) and grams (g).

In human HD, a lower body mass index is a symptom in the prodromal phase of the disease^31^. In this study, we saw equivalent patterns of body weight loss in the heterozygous mice (Fig. 5A). To investigate if this body weight loss is driven by a reduction in lean or fat mass, mice underwent Echo MRI scanning. These experiments revealed that whilst these mice have a slight increase in overall lean mass when adjusted for body weight, the weight loss in the heterozygous mice is largely driven by a reduction in fat mass (Fig. 5C + D). In addition to a reduction in body weight, it was also observed that heterozygous mice had lower brain weights than wild-type mice (Fig. 5B), in line with brain atrophy seen in human HD^32^.

### Huntingtin aggregation occurs in later stages of disease progression and is variable across brain regions

**Fig. 6:**
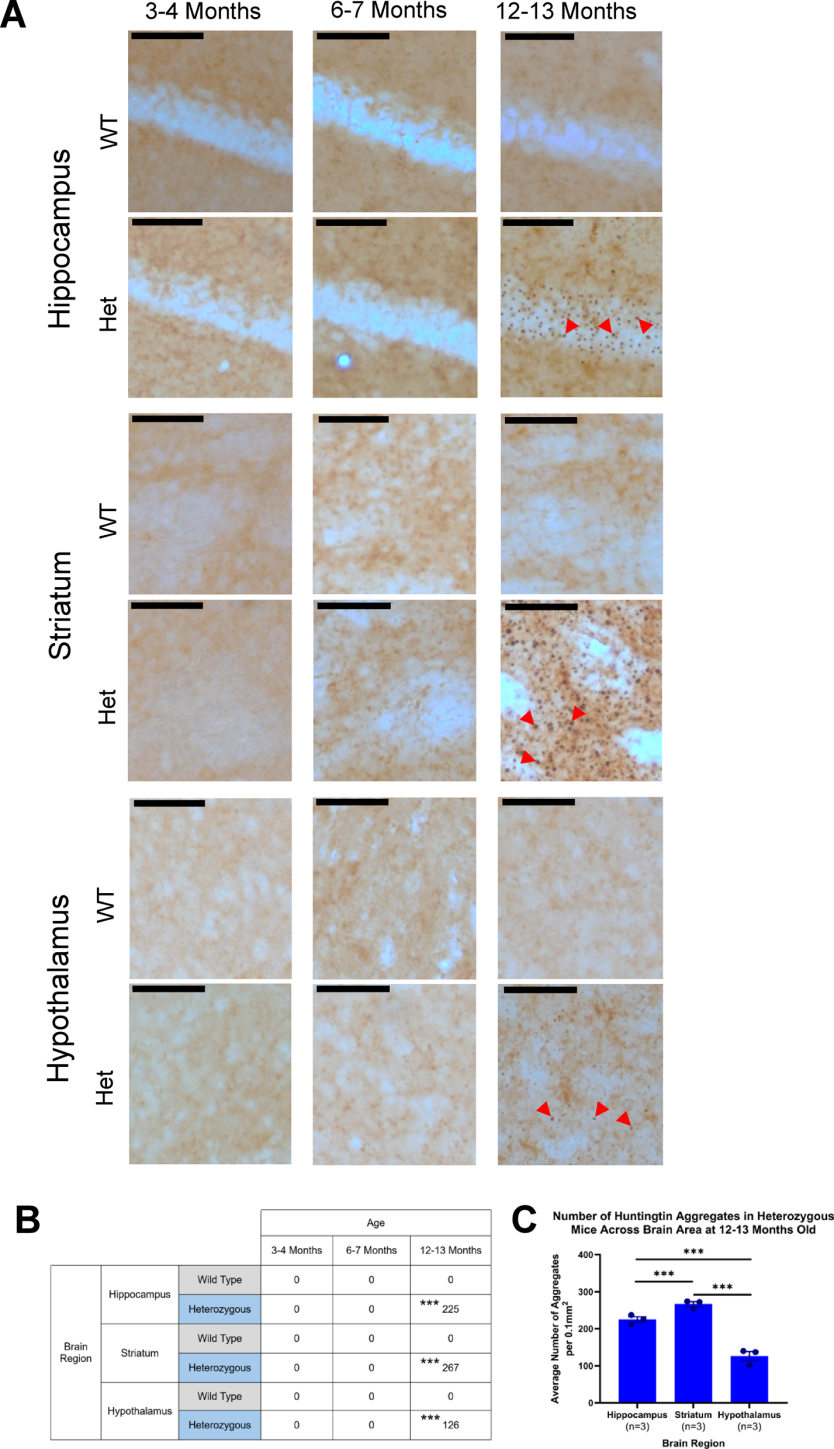
**Huntingtin aggregation pathology in hippocampus**,** striatum and hypothalamus of wild-type and heterozygous zQ175 mice using mEM48 antibody.** (**A**) Representative images of mutant huntingtin aggregate staining in WT and Het mice aged 3–4, 6–7 and 12–13 months in hippocampus, striatum and hypothalamus. WT mice aged 3–4, 6–7 and 12–13 months do not have positive staining for huntingtin aggregates. Het mice at 3–4 and 6–7 months also have no detectable aggregates. Het mice aged 12–13 months have aggregates in the hippocampus, striatum and hypothalamus. Arrows highlight example aggregates in sections. (**B**) Average number of aggregates per 0.1mm^2^ in WT and Het mice aged 3–4, 6–7 and 12–13 months in hippocampus, striatum and hypothalamus. There were no significant differences between WT and Het for hippocampus, striatum, and hypothalamus at 3–4 and 6–7 months. At 12–13 months, significant differences were found between WT and Het for hippocampus, striatum, and hypothalamus. For WT mice, no significant differences were detected for comparisons between any ages or regions. There were also no significant differences in Het mice at 3–4 vs. 6–7 months for hippocampus, striatum, or hypothalamus. There were also no differences between regions in Het mice when compared at 3–4 or 6–7 months. Significant differences were found in Het mice in hippocampus at 3–4 vs. 12–13 months and 6–7 vs. 12–13 months, striatum at 3–4 vs. 12–13 months and 6–7 vs. 12–13 months, and hypothalamus at 3–4 vs. 12–13 months and 6–7 vs. 12–13 months. There were also significant differences in Het mice at 12–13 months when different regions were compared (hippocampus vs. striatum, hippocampus vs. hypothalamus, striatum vs. hypothalamus). (**C**) Comparison of aggregates per 0.1mm^2^ in Het mice aged 12–13 months in hippocampus, striatum and hypothalamus. Multiple comparisons as stated above in (B). Striatum showed highest number of aggregates, followed by hippocampus, and hypothalamus with the least. Three-way ANOVA with multiple comparisons and Bonferroni corrections where appropriate. Data are mean ± SEM. *p < 0.05, **p < 0.01, ***p < 0.001. All groups *n* = 3. Wild-type (WT), heterozygous (Het). Scale bar = 100 μm.

Huntingtin aggregates are a pathological hallmark of HD and often associated with disease progression in both human HD and mouse models^33^. To investigate if aggregates were present in brain regions relevant to disease symptoms, the striatum, hypothalamus, and hippocampus were analysed to assess regions related to motor, metabolic and cognitive functions, respectively (Fig. 6). Through staining for mutant and aggregated huntingtin (mHTT) in the brains of heterozygous mice, it was shown that aggregates were not detectable at 3–4 or 6–7 months old in any of the regions examined. However, by 12–13 months old, aggregates were detected in the striatum, hypothalamus, and hippocampus (Fig. 6A + B). The presence of aggregates in the striatum and hippocampus coincided with motor and memory deficits, respectively. However, changes in body weight (6 months onwards) occur before the appearance of huntingtin aggregates in the hypothalamus. Further analyses comparing the different regions showed that the striatum had the most aggregates, followed by the hippocampus then the hypothalamus (Fig. 6C).

### Hippocampal huntingtin aggregation coincides with cognitive deficits and differs across subregions

**Fig. 7:**
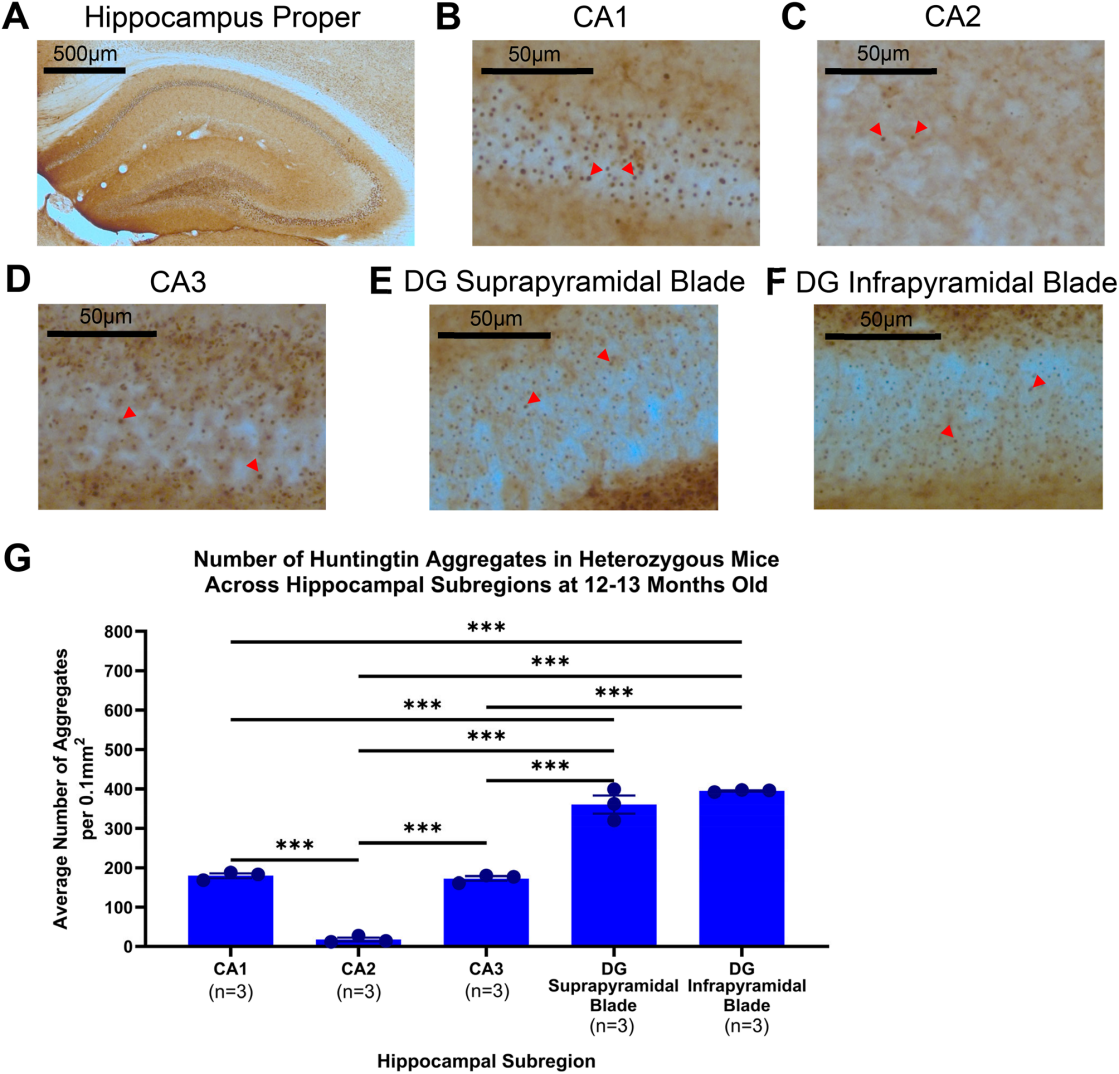
Huntingtin aggregation pathology in hippocampal subregions of 12–13-month-old heterozygous zQ175 mice. Representative image of mutant huntingtin aggregate staining in 12–13 month heterozygous mice in (**A**) Hippocampus proper (**B**) CA1 region (**C**) CA2 region (**D**) CA3 (**E**) DG suprapyramidal blade (**F**) DG infrapyramidal blade (**G**) Comparison of the average number of aggregates per 0.1mm^2^ in Het mice aged 12–13 months in hippocampal subregions. Notably, the CA2 had very few aggregates and significantly less than all other hippocampal subregions. One-way ANOVA with multiple comparisons and Bonferroni corrections where appropriate. Data are mean ± SEM. *p < 0.05, **p < 0.01, ***p < 0.001. All groups *n* = 3. Wild-type (WT), heterozygous (Het), Cornu ammonis (CA) and dentate gyrus (DG).

Aggregates were detected in the hippocampus at 12–13 months old, alongside the development of deficits in hippocampal-dependent long-term and spatial memory tasks. Due to a particular interest to understand aspects of the zQ175 mouse in relation to cognition, aggregates in the hippocampus were analysed in further detail to investigate if there were subregion disparities. The pyramidal layers of the cornu Ammonis (CA) regions including CA1, CA2 and CA3 were analysed, as well as the granule cell layers of the suprapyramidal and infrapyramidal blades of the dentate gyrus (DG) (Fig. 7A-F). These analyses revealed that the CA1 and CA3 regions have comparable numbers of aggregates (Fig. 7G). The dentate gyrus (DG) suprapyramidal and infrapyramidal blades were also comparable in number of aggregates, however, both had significantly more than the CA1 and CA3 regions. Notably, the CA2 had significantly less than all the other hippocampal subregions.

### Hippocampal long-term potentiation (LTP) deficits occur early in the disease progression and prior to the emergence of symptoms

**Fig. 8:**
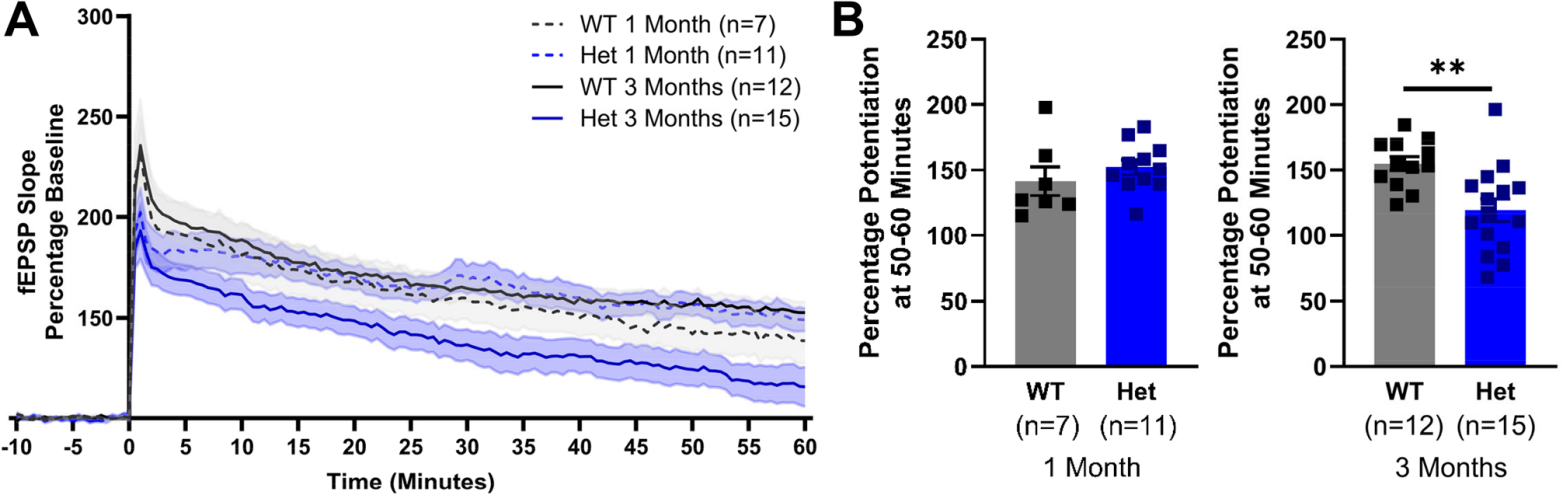
Long-term potentiation in 1-month and 3-month-old wild-type and heterozygous zQ175 mice. LTP is impaired in 3-month-old Het mice. (**A**) Delivery of a 4-TBS caused an initial increase in the slope of the fEPSPs in all mice. The responses of 3 month WT, 1 month WT and 1 month Het stabilize above baseline after 30 min, an effect well maintained at 50–60 min. In 3-month-old Het mice, the initial induction of LTP is reduced compared to 3 month WT. The 3 month Het response gradually decreases to below the 3 month WT response at 50–60 min after 4-TBS. (**B**) Average % potentiation above baseline observed 50–60 min after 4-TBS. LTP of 3 month Het is significantly impaired compared to 3 month WT. There is no difference in % potentiation between 1 month Het and 1 month WT. One-way ANOVA. Data are mean ± SEM. WT vs. Het *p < 0.05, **p < 0.01, ***p < 0.001. 3 month WT *n* = 12, 3 month Het *n* = 15, 1 month WT *n* = 7, 1 month Het *n* = 11. Wild-type (WT), heterozygous (Het), long-term potentiation (LTP), 4-pulse theta-burst stimulation (4-TBS) and field excitatory postsynaptic potentials (fEPSPs).

Hippocampal LTP is widely considered as a neural correlate of learning and memory^17^. Here, LTP was initially determined in 3 month wild-type and heterozygous mice, a developmental age chosen to correspond with the first time-point of behavioural testing. At 3 months old, no deficits were observed in the performance of long-term or spatial memory tasks in the heterozygous mice. However, despite evidence that these behaviours are dependent on the hippocampus^34,35^, the heterozygous mice presented with significantly impaired LTP (Fig. 8). For wild-type mice the 4-TBS produced an increased LTP when compared to that of baseline fEPSPs obtained prior to delivery of the 4-TBS. By comparison, the magnitude of LTP obtained from heterozygous littermates was significantly reduced. To investigate if LTP was intact at an earlier age, 1-month-old mice were tested and there was no difference between wild-type and heterozygous mice. The data demonstrate that synaptic plasticity is impaired prior to the formation of aggregates and deficits in memory tasks, therefore, LTP may be a more sensitive measure of hippocampal deterioration in HD.

## Discussion

In our study of the zQ175 mouse, we present data characterising key aspects of HD, including the less studied cognitive, psychiatric and metabolic manifestations, as well as classical disease symptoms. Cognitive and psychiatric symptoms are some of the first to affect HD patients and are recognised as debilitating^7–9,25^. In this study, deficits in visuospatial attention were detected early, at approximately 3–4 months, while heterozygous mice were less impulsive than their wild-type littermates. Subtle motor slowing was also observed in this task. These data are consistent with the impact of impaired striatal dysfunction on performance in the 5CSRTT^20,36^ and are similar to profiles of impairments reported in other KI HD mutant mice including heterozygous HdhQ111 and homozygous HdhQ92^21,22^. Additionally, visuospatial deficits are some of the earliest to emerge in HD patients^23,24^. This aligns with our studies, as whilst we detected deficits in the 5CSRTT task at 3–4 months, we did not however detect long-term and spatial memory impairments until 12–13 months. By this time, we also observe the onset of more overt motor impairments. This implies that cognition dependent on the hippocampus is acutely affected once the classical motor symptoms have been realised. This concurrence corresponds with human HD, where MCI and dementia are prevalent in patients when motor symptoms manifest^8,37^. We do not, however, observe any hippocampal-associated cognitive impairments in the mouse equivalent of the human prodromal phase of the disease, nor any changes in performance in the short-term memory task, as is seen in human HD^8,38,39^. Hippocampal-associated deficits have been detected much sooner in other mouse models, with the R6/2 showing spatial memory deficits by 7 weeks old^40^, although this would be expected, due to the accelerated nature of the disease progression of this model. There is also evidence YAC128 mice have spatial memory deficits by 8 months old, with some reports even by 2 months^41,42^. The KI lines are not as explicit, with the HdhQ92 displaying mild spatial memory deficits, although these experiments were carried out in homozygous mice^43^. Additionally, researchers have reported attentional and executive function alterations in the HdhQ111 mouse at 18 months old, although these are not progressive^44^, and the CAG 140 KI mouse has no clear reported cognitive deficits^10^. Whilst there have been limited studies looking at hippocampal-associated memory in the zQ175 mouse, there is prior evidence that deficits occur in a 24-hour NOR protocol in both heterozygous and homozygous zQ175 mice^45^. This study did not assess if the mice could do this task prior to 12 months old, however, we have shown in our own study that there were no deficits detected at earlier time points. Taken together, the zQ175 develops cognitive deficits in a time-relevant manner, in line with symptoms seen in human HD. Compared to other KI models, the zQ175 develops cognitive deficits more quickly, although not as fast as seen in many transgenic models. Whilst this may not be as useful for therapeutic studies, the zQ175 provides a suitable length of time to investigate the prodromal stage of HD and may be particularly useful to investigate early changes in cellular mechanisms.

Anxiety is often reported as a symptom in human HD, however, in contrast, this study showed that zQ175 mice developed an anxiolytic-like phenotype as the disease progressed. This apparent disconnect may be due to patients with HD often reporting the source of their anxiety as their disease prognosis^46^, which would not apply to mice. The anxiolytic-like behaviour observed may be a manifestation of early cognitive disruptions, which implicate not only dysfunction in the amygdala, but also the hippocampus^47,48^. Anxiolytic-like phenotypes have also been found in the R6/2 mice from as early as 8 weeks old^49,50^, however, there have been conflicting reports in the HdhQ111 and evidence of increase anxiety in the CAG 140 KI mouse from 6 weeks and FDNQ175 from 6 months^16,51–53^. More studies are required to establish why such differences exist and investigate the impact of factors such as background strain. In our study the changes in anxiolytic-like behaviours are the earliest neuropsychiatric changes we detected in heterozygous zQ175 mice. They occur prior to the onset of overt motor symptoms, in line with human HD where psychiatric changes occur in the prodromal phase of the disease^7^.

Motor impairments have classically defined HD in humans, with these symptoms being the most widely recognised. As the disease progresses, motor impairments including dystonia, rigidity and bradykinesia, become more prominent^5^. The zQ175 mouse recapitulates this aspect of human HD, showing subtle motor slowing as early as 3–4 months on the operant 5CSRTT and the open field test, while more overt motor impairments in both the vertical pole and rotarod tests emerged after 12 months. Deficits in the vertical pole and rotarod tasks indicate that these later motor impairments are associated with endurance. Additionally, the downward trend in the fixed speed rotarod data for heterozygous mice at 6–7 months, suggests these impairments are progressive. Measurements of motor ability are some of the most widely investigated characteristics of HD mouse models, making them a useful comparison metric. The R6/2 mouse has clear motor deficits by 8–12 weeks, however, there have also been reports of changes as early as 5–6 weeks old^54,55^. This is unsurprising due to the known accelerated nature of the R6/2 disease progression. BACHD and YAC128 mice also show early motor deficits, by 2 months, which are progressive, and performance by 12 months is severely impaired^42,56–58^. In the KI models, there is little evidence of any prominent motor deficits in the HdhQ92^43^, the HdhQ111 shows some subtle impairments from 9 to 12 months old^59^, and the CAG 140 KI has varied presentations of motor symptoms, from mild gait anomalies at 12 months to rotarod impairments at 4 months old^13,51^. Studies using the zQ175dn line did not detect motor deficits without readjusting for body weight differences^15^. In our study we detected motor deficits both before and after adjusting for weight. Although it should be noted that we also performed a fixed speed protocol in addition to the accelerating speed protocol, which may be more sensitive. In summary, we found the zQ175 mouse showed a progressive and robust motor phenotype, making it suitable for studies wishing to investigate this aspect of HD.

Unintended catabolic weight loss is one of the most common, and earliest, occurring symptoms in human HD^6^. The zQ175 mouse emulates this disease characteristic well, showing impeded growth curves and reductions in fat mass. It is still not known what this weight loss is attributed to, however, human studies have shown that grey matter alterations in the hypothalamus occur prior to clinical diagnosis^60^. Furthermore, there is evidence that the weight loss could be driven by alterations in peripheral signals, such as reduced ghrelin activity^61^. Interestingly, studies have shown that the expanded CAG repeats are linked to the development of diabetes in both mouse models and HD patients^62^ and there is evidence of altered brain glucose usage^63,64^. Further experiments have shown that this may be a result of dysfunctional astrocyte-to-neuron signalling^65^. Considering the complex relationship between altered metabolism and neurodegeneration in conditions not typically recognised as metabolic diseases, such as HD, understanding how mutant huntingtin leads to metabolic dysfunction may help allude to mechanisms of this disease and potential new therapeutic targets.

Alongside cognitive, psychiatric, motor, and metabolic symptoms, we observed a reduction in brain volume and an increase in mutant huntingtin protein, which accumulates, and forms aggregates in the brain. There is dispute over how aggregates contribute to cell survival, with some researchers believing aggregates are toxic, cause neuronal vulnerability and induce cell death, whilst others believe they may have a protective role^33,66^. Nonetheless, they are a hallmark of human HD and typically detected across brain regions^67^. It is difficult to estimate when these aggregates start forming in humans, as confirmation can only occur post-mortem, however mouse models can be utilised to better understand the pathology progression. In this study, aggregates were not detected until 12–13 months, when wide-spread staining across regions was observed. Interestingly, it has been suggested that phenotypic changes are not dependent on aggregated deposition. This has been seen in both rat models of HD^68^ and the HdhQ92 mouse where the 5CSRTT task showed similar operant deficits to our own study at 3–4 months old and observed these deficits to be independent of aggregate pathology^69^. However, a study using an alternative assays, antibodies and antigen retrieval techniques has suggested that subtle mHTT aggregation could be observed as early as 1–2 months in zQ175 in striatal and hippocampal regions^70^. This suggests that neurons are sensitive to subtle mHTT aggregation which can disrupt their function before the occurrence of large inclusion bodies normally associated with HD pathology. Consistent with this literature, we observe diffuse mHTT staining in the striatum at 3 months of age using the S830 antibody (Figure S5). We also observed diffuse mHTT in the hippocampus at 3 months of age, and more punctate nuclear aggregates were evident in the striatum, hippocampus and hypothalamus by 12 months of age. Although both mEM48 and S830 have been shown to label diffuse and aggregated mHTT^71^, in our hands differences in mHTT expression were evident in the 3 month tissue depending on the specific antibody used, with S830 being more sensitive to the early diffuse staining than mEM48. Indeed, S830 has been used recently to reveal mHTT expression in the zQ175 model as early as 1 month of age^70^.

There has been little investigation into aggregate deposition in the hypothalamus of mouse models, despite the consistent metabolic alterations. However, in this study, it was demonstrated that there were high numbers of aggregates present in the hypothalamus, albeit less than in the striatum. Furthermore, despite cognitive disturbances being reported early in human HD, and prior to motor symptoms, there has been limited exploration into aggregate formation in the hippocampus. In this study, aggregate deposition in hippocampal subregions was examined to explore subregion variability. Large numbers of aggregates were observed in the CA1, CA3 and DG. Furthermore, the highest number of aggregates were found in the DG, a region of unique plasticity where adult neurogenesis occurs^72^. Intriguingly, the CA2 region had far fewer aggregates than the CA1, CA3 and DG. Whilst this may be surprising due to the widespread aggregation seen across the hippocampus, the CA2 is often shown to be anomalous to other CA regions in both form and function, as studies investigating the CA2 have revealed, unlike other hippocampal subregions, it is resistant to cell vulnerability and death^73,74^. These findings highlight the different vulnerabilities of hippocampal subregions in neurodegenerative diseases.

Our study found that LTP was diminished in 3 month, heterozygous zQ175 mice. This perturbation was the earliest change we observed in this model and, interestingly, this did not coincide with deficits in long-term and spatial memory tasks. This suggests that impaired LTP is an early indicator of disease onset, which arises independently of aggregate formation and does not compromise memory immediately. Whilst there are currently no other studies investigating electrophysiological changes in the hippocampus of the zQ175 mice, there have been studies which have found evidence of disruption in circuit activity in other brain regions including the cortex, striatum, basal ganglia and subthalamic nucleus in zQ175 mice aged 2 months and older^75–78^. Our data showed that deficits in hippocampal synaptic plasticity developed with age, as 1 month heterozygous zQ175 mice had normal LTP. This indicates that this form of synaptic plasticity deteriorated as the disease progressed. In other studies, it was shown that 2 month HdhQ111 mice had impaired hippocampal LTP, alongside a reduction of brain-derived neurotrophic factor (BDNF) in the hippocampus^79^. Additionally, there was a reduction of dendritic spine density in hippocampal slices of HdhQ111 mice after a TBS, similar to the protocol used in our study. Importantly, BDNF restored hippocampal LTP and the number of dendritic spines after TBS in HdhQ111 mice, suggesting that deficits in actin polymerisation resulting in reduced spine density post-TBS caused by a reduction of BDNF underlies the LTP deficits found early on in HD mouse models^79^. Additional studies in HD mouse models have shown that defective BDNF signalling through the tyrosine receptor kinase B (TrkB) pathway contributes to dysregulated AMPA receptor trafficking and surface diffusion in hippocampal neurons^80^. Collectively these data show that impairments in LTP and underlying regulatory mechanisms caused by HD mutations occur months prior to behavioural deficits and huntingtin aggregate deposition. These studies are helping to unravel the underlying mechanisms driving HD.

Overall, the zQ175 mouse model appears to have an accelerated and more pronounced phenotype compared to the CAG 140 KI parent line. This supports the idea that the more CAG repeats, the more severe the phenotype. However, there is also evidence that expanding the CAG repeats beyond 185 in the zQ175 mouse model does not accelerate the onset of molecular and neuropathological phenotypes^81^. Further evidence of this anomaly in HD mouse models is in the R6 lines, where there is variable CAG repeat size from approximately 40 all the way up to 600. Whilst an increase in severity is seen up to 200 CAG repeats, once the repeats reach above 200, symptoms start decreasing^82–84^. One explanation may be that the protein is too large to cross into the nuclei *via* the nuclear pore, where it can induce neurodegeneration^85,86^. Mouse models also require many more CAG repeats than humans for symptoms to occur, which indicates that mice have a higher threshold for polyglutamine expansions. This difference is despite the endogenous number of CAG repeats in mice being between 2 and 4, compared to healthy humans which have between 19 and 35 CAG repeats^11^. According to evidence in the literature, the number of CAG repeats that induces a HD phenotype in mice is approximately 70–80 CAG repeats^86,87^. The resilience of mice to CAG expansions compared to humans raises interesting questions about what we can learn from mice and the cellular mechanisms they have for clearing aggregates across a range of neurodegenerative diseases. The wide range of HD mouse models can help address why the CAG expansion is damaging to cells and whether there are mechanisms, beyond the protein, which can cause issues.

In our study there are largely no behavioural differences between male and female heterozygous zQ175 mice. We found no statical interactions between genotype and sex, except in the 5CSRTT (reaction time) and in the open field (maximum speed) at 12–13 months (Supplementary Figure S2, Supplementary Figure S4, Supplementary Table S3), indicating that male zQ175 mice may be more affected by locomotor HD symptoms than females, although no statical interactions between genotype and sex were detected in the rotarod or vertical pole tasks (Fig. 4, Supplementary Table S3). Previous studies examining sex differences in heterozygous zQ175 mice found that males exhibited decreased locomotor activity, whilst females did not^14^. In the zQ175dn model, motor and cognitive impairments were detected in heterozygous males but not females^15^. Furthermore, male FDNQ175 mice were found to be more vulnerable to locomotor decline^88^. Together, this suggests males in Q175 mouse lines are more susceptible to HD symptom manifestation. In humans, there is inconsistent evidence of genetic penetrance and prevalence of HD in men and women^89^. Recent studies suggest that oestrogen may have a protective effect in HD^90,91^, although it has also been reported than there is an increased prevalence and symptom severity in women, specifically with motor abilities and depression^92,93^.

A key aspect of a useful mouse model is the reproducibility of data across different research groups. Our data typically agrees with previous studies investigating the zQ175 mouse, with similar observations in weight loss trajectory and overt motor symptom onset^14,94^. There were some discrepancies, with other studies detecting aggregates earlier, from 4 to 8 months in the striatum and cortex respectively, whilst we only detected aggregates at 12 months, although diffuse mHTT could be seen from 3 months^95^. Cognitive outputs, particularly related to memory, have not been well characterised, however, in our study, we show that there were no deficits in the short-term novel object task, whilst impairments were detected in visuospatial attention, long-term novel object and spatial memory tasks. This highlights that the development of cognitive impairments in the zQ175 mouse may be dependent on brain region dependency and task difficulty^14,94^.

The zQ175 mouse model mirrors certain aspects of the human disease. The zQ175 mouse shows classical characteristics of HD, including brain pathology and a robust motor phenotype. Additionally, these mice have a metabolic phenotype which make it appropriate for studying metabolic disturbances in HD. The temporal dissociation of psychiatric and metabolic phenotypes with cognitive and motor deficits allows for testing of prodromal disease phenotype and possible rescue prior to the debilitating motor impairments. Finally, the zQ175 mice would be suitable for modelling the human disease progression in longitudinal studies and could be particularly informative in studies investigating the stages preluding the development of cognitive symptoms.

## Supplementary Information

Below is the link to the electronic supplementary material.

Supplementary Material 1

Supplementary Material 2

Supplementary Material 3

Supplementary Material 4

## Data Availability

The datasets generated during and/or analysed during the current study are available from the corresponding author on reasonable request.
