# Supplementary material for: Development of cognitive, motor, metabolic, and mutant huntingtin aggregation in the zQ175 mouse model of Huntington’s disease
Source: Sci Rep. 2025 Oct 3;15:34563. doi: 10.1038/s41598-025-17956-5 (PMC12494737; doi:10.1038/s41598-025-17956-5)
Supplement: Supplementary file 1 — Supplementary Material 1 [file 41598_2025_17956_MOESM1_ESM.pdf]

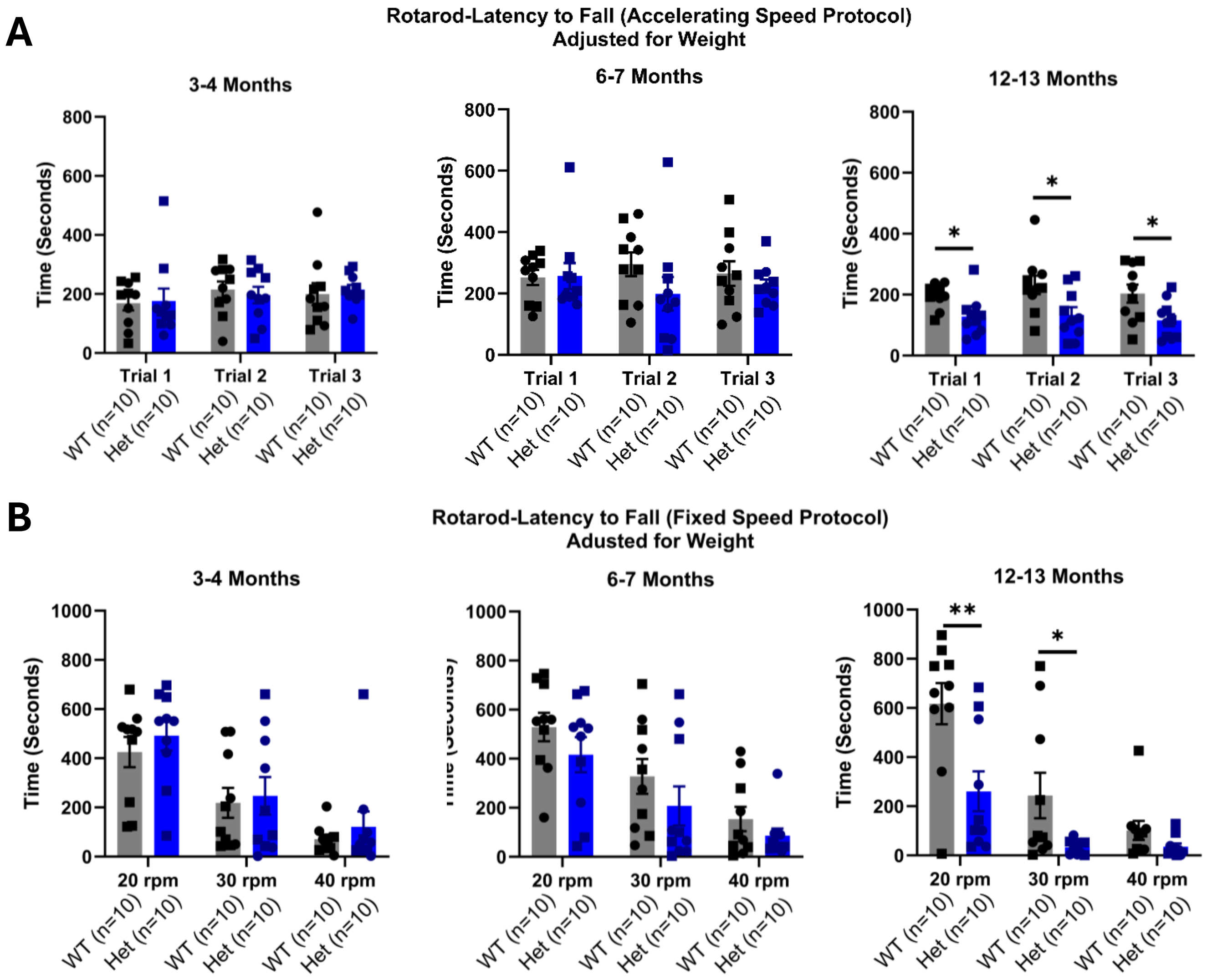

Figure S1. Rotarod in wild-type and heterozygous zQ175 mice adjusted for weight. (A) Latency to fall from rotarod with accelerating protocol adjusted for weight. There were no differences at 3-4 or 6-7 months between WT and Het mice. At 12-13 months there were significant difference between WT and Het mice. (B) Latency to fall from rotarod with fixed speed protocol adjusted for weight. At 3-4 or 6-7 months there were no differences between WT and Het mice at 20rpm, 30rpm or 40rpm. At 12-13 months there were differences between WT and Het mice at 20rpm and 30rpm but not 40rpm. Two-way repeated measures ANOVA with pairwise comparisons and Greenhouse–Geisser and Bonferroni corrections where appropriate. Data are mean ± SEM. Symbols are square (male) and circle (female). WT vs Het *$p < 0.05$, **$p < 0.01$, ***$p < 0.001$; All groups n=10. Wild-type (WT), heterozygous (Het) and rotations per minute (rpm).

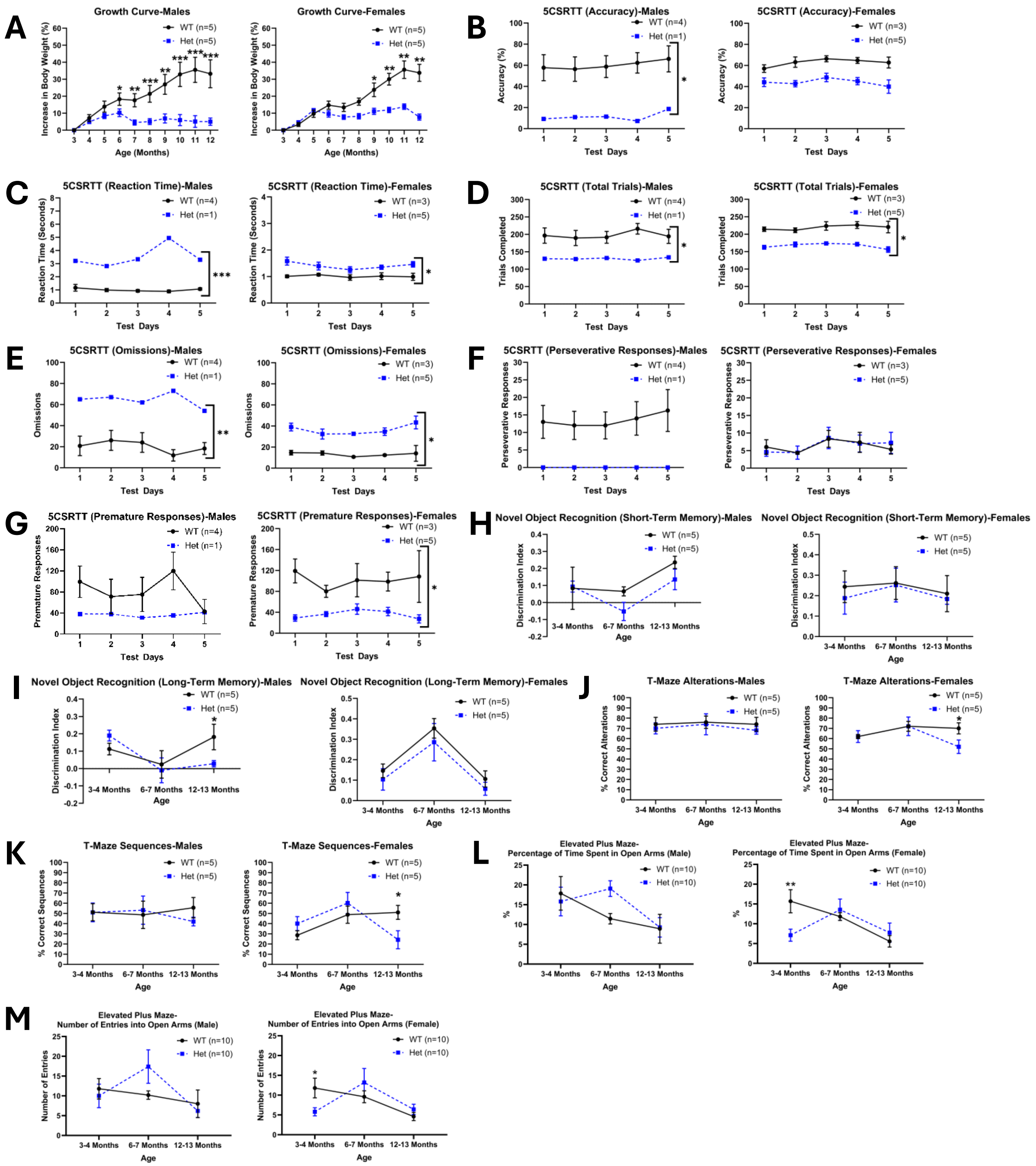

Supplementary Figure S2. Behavioural tasks presented in line graphs split by sex for wild-type and heterozygous zQ175 mice. (A) Body weight (B) 5CSRTT (response accuracy) (C) 5CSRTT (reaction time) (D) 5CSRTT (trials completed) (E) 5CSRTT (omissions) (F) 5CSRTT (perseverative responses) (G) 5CSRTT (premature responses) (H) Short-term memory novel object recognition task graphs. (I) Long-term memory novel object recognition task graphs. (J) T-Maze alternations graphs. (K) T-Maze sequences graph. (L) Elevated plus maze (percentage of time spend in open arms) (M) Elevated plus maze (number of entries into open arms). Data are mean ± SEM. WT vs Het $^*p < 0.05$, $^{**}p < 0.01$, $^{***}p < 0.001$. Wild-type (WT) and heterozygous (Het).

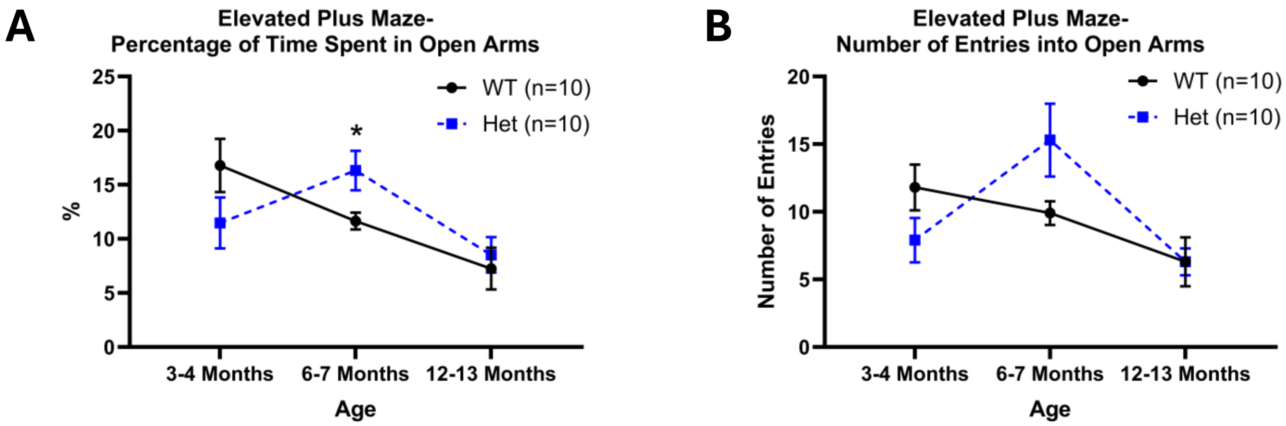

Figure S3. Elevated plus maze in wild-type and heterozygous zQ175 mice. (A) Percentage of time spent in open arms. There were no differences at 3-4 or 12-13 months between WT and Het mice. At 6-7 months, Het mice spent more time in the open arms. (B) Number of entries into open arms. There were no significant differences between WT and Het mice across ages. Two-way repeated measures ANOVA with pairwise comparisons and Greenhouse–Geisser and Bonferroni corrections where appropriate. Data are mean ± SEM. WT vs Het *$p < 0.05$, **$p < 0.01$, ***$p < 0.001$; All groups n=10. Wild-type (WT) and heterozygous (Het).

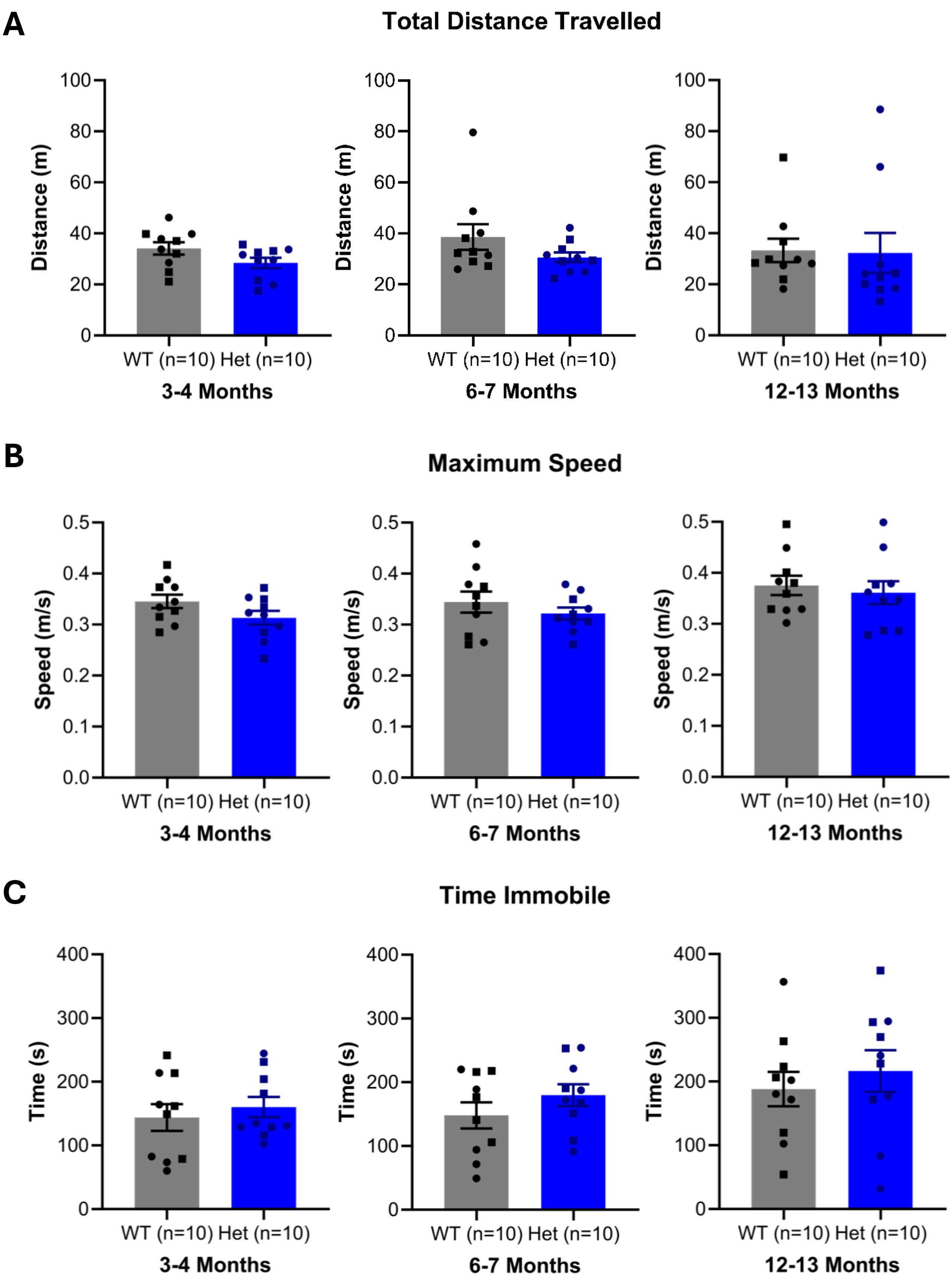

Supplementary Figure S4. Additional measurements for the open field task in wild-type and heterozygous zQ175 mice. (A) Total distance travelled. There were no differences between WT and Het mice at any age. (B) Maximum speed. There were no differences between WT and Het mice at any age. (C) Time Immobile. There were no differences between WT and Het mice at any age. One-way ANOVA. Data are mean ± SEM. Symbols are square (male) and circle (female). WT vs Het $*p < 0.05$, $**p < 0.01$, $***p < 0.001$; All groups n=10. Wild-type (WT) and heterozygous (Het).

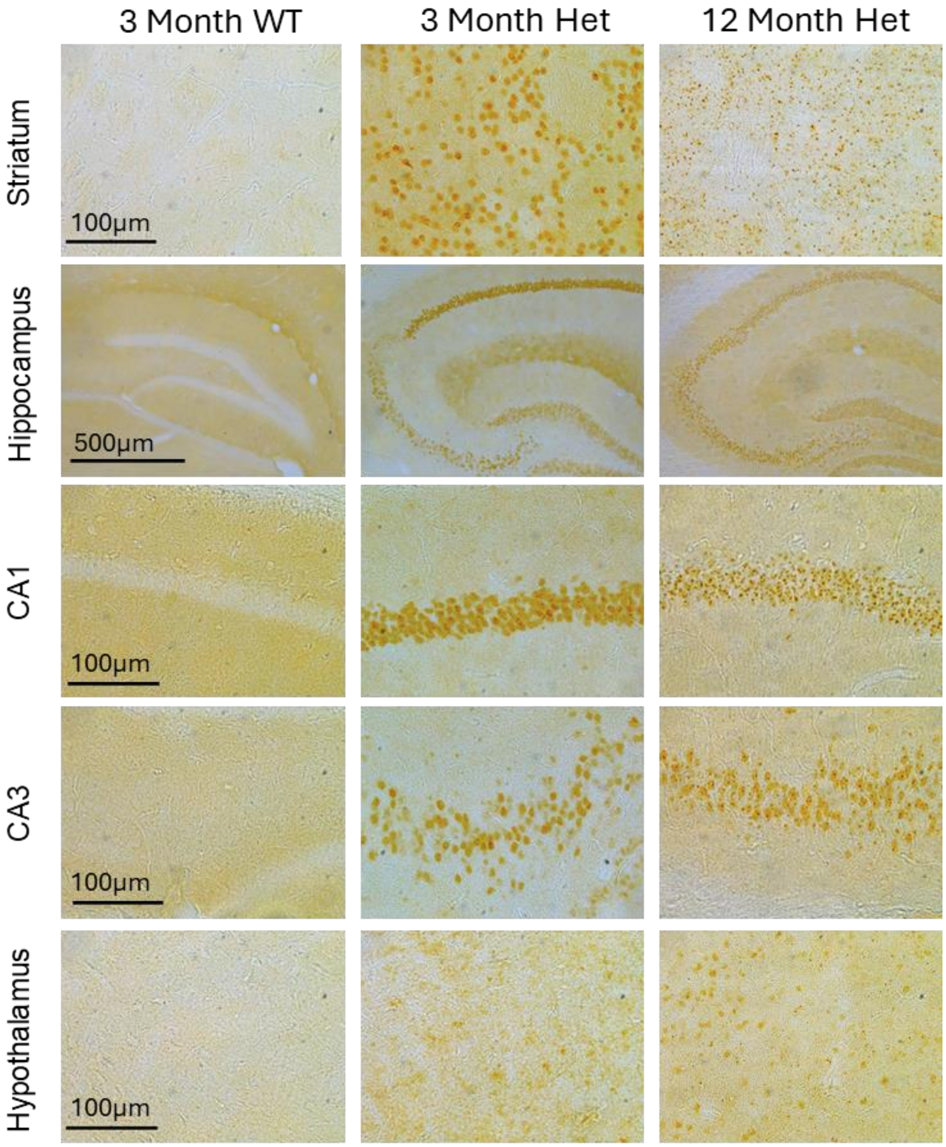

Figure S5. Huntingtin pathology in hippocampus, striatum and hypothalamus of wild-type and heterozygous zQ175 mice using S830 antibody. (A) Representative images of mutant huntingtin aggregate staining in WT mice aged 3-4 months and Het mice aged 3-4 and 12-13 months in hippocampus, striatum and hypothalamus. WT mice did not have positive staining for huntingtin aggregates in any brain regions. Het mice at 3-4 months had detectable diffuse staining in the striatum and hippocampus. By 12-13 months of age, more punctate aggregates were evident in the striatum and hippocampus, and some diffuse and punctate staining were evident in the hypothalamus. Het mice aged 12-13 months had aggregates in the hippocampus, striatum and hypothalamus. Scale bars for striatum, CA1, CA3, and hypothalamus are 100um (taken with 40x objective). Scale bar for the hippocampus is 500um (10x objective).
